# Genome-wide diversity and MHC characterisation in a critically endangered freshwater turtle susceptible to disease

**DOI:** 10.1007/s00251-025-01378-8

**Published:** 2025-05-06

**Authors:** Holly V. Nelson, Luke Silver, Toby G. L. Kovacs, Elspeth A. McLennan, Arthur Georges, Jane L. DeGabriel, Carolyn J. Hogg, Katherine Belov

**Affiliations:** 1 School of Life and Environmental Sciences, The University of Sydney, Sydney, NSW 2006 Australia; 2 Australian Research Council Centre of Excellence for Innovations in Peptide and Protein Science Science, The University of Sydney, Sydney, NSW 2006 Australia; 3 Institute for Applied Ecology, University of Canberra, Bruce, ACT 2617 Australia; 4 NSW Department of Climate Change, the Environment,, Energy and Water, Parramatta, NSW 2150 Australia

**Keywords:** Conservation genomics, Immune genes, Bellinger River turtle, Major histocompatibility complex, Whole-genome re-sequencing

## Abstract

Small, isolated populations are often vulnerable to increased inbreeding and genetic drift, both of which elevate the risk of extinction. The Bellinger River turtle (*Myuchelys georgesi*) is a critically endangered species endemic to a single river catchment in New South Wales, Australia. The only extant wild population, along with the breeding program, face significant threats from viral outbreaks, most notably a nidovirus outbreak in 2015 that led to a 90% population decline. To enhance our understanding of genomic characteristics in the species, including genome-wide and functional gene diversity, we re-sequenced, assembled, and analysed 31 re-sequenced genomes for pure *M. georgesi* (*N* = 31). We manually annotated the major histocompatibility complex (MHC), identifying five MHC class I and ten MHC class II genes and investigated genetic diversity across both classes in *M. georgesi*. Our results showed that genome-wide diversity is critically low in pure *M. georgesi*, contexualised through comparison with opportunistically sampled backcross animals—offspring of F1 hybrids (*M. georgesi* × *Emydura macquarii*) backcrossed to pure *M. georgesi* (*N* = 4). However, the variation observed within the core MHC region of pure *M. georgesi*, extending across scaffold 10, exceeded that of all other macrochromosomes. Additionally, no significant short-term changes in either genome-wide or immunogenetic diversity were detected following the 2015 nidovirus outbreak (before; *N* = 19, after; *N* = 12). Demographic history reconstructions indicated a sustained, long-term decline in effective population size since the last interglacial period, accompanied by more recent steep declines. These patterns suggested that prolonged isolation and reduced population size have significantly influenced the dynamics of genome-wide diversity. It is likely that contemporary stressors, including the recent nidovirus outbreak, are acting on an already genetically depleted population. This study offers new insights into genome-wide and immune gene diversity, including immune gene annotation data with broader implications for testudines. These findings provide crucial information to support future management strategies for the species.

**Supplementary Information:**

The online version contains supplementary material available at 10.1007/s00251-025-01378-8.

## Introduction

The emergence of novel infectious diseases is currently a major threat faced by species of conservation concern (Daszak et al. 2000; Smith et al. 2009). Warming climates, pollution, and introduced species that harbour invasive pathogens are facilitating the spread of disease across wildlife populations (Anderson et al. 2004). Managing declining populations in the presence of infectious disease, in addition to other anthropogenic threats, is of growing conservation concern. Understanding the mechanisms of resilience and resistance, and the degree to which populations can adapt to change is a key step towards preventing extinctions (Auteri and Knowles 2020). Another important aspect of conservation management is maintaining the adaptive potential of a species. Adaptive potential is the ability of a population to adapt to changing environmental conditions, such as disease, habitat modifications, and climate change (Hoffmann et al. 2017; Holderegger et al. 2006). Species’ recovery programs are increasingly using genomic data to understand the adaptive potential of populations through functional gene analyses (McLennan et al. 2024), with growing emphasis on how managers can maintain or potentially increase adaptive potential in wild populations and conservation breeding programs (Farquharson et al. 2022). Advances in sequencing technologies and bioinformatic tools, and reductions in sequencing costs, have made whole-genome data more accessible for threatened species recovery programs. The combination of high-quality reference genomes with whole-genome sequencing data facilitates high-resolution analyses of neutral genetic variation, while also enabling the reconstruction of historical demographic trends and characterisation of functional gene families (Theissinger et al., 2023). For example, Magid et al. (2022) used 66 re-sequenced genomes to investigate toll-like receptors (TLR) immune gene diversity in shore plovers (*Thinornis novaeseelandiae*) and found low levels of diversity.

The major histocompatibility complex (MHC) is a crucial gene family within the adaptive immune system, primarily responsible for antigen presentation (Piertney and Oliver 2006). MHC molecules bind and present pathogen-derived peptides to T cells, facilitating the recognition and initiation of immune responses to pathogens and ensuring the specificity of adaptive immunity (Neefjes et al. 2011). MHC genes are broadly classified into classical and non-classical categories. Classical MHC genes, for example MHC class Ia and class II, are highly polymorphic and play a direct role in antigen presentation, influencing immune recognition and pathogen resistance (Le Bouteiller and Lenfant 1996). MHC class Ia and class II molecules are widely expressed across various tissues but are most prominent in antigen-presenting cells (Neefjes et al. 2011). In contrast, non-classical MHC genes (e.g., MHC class Ib, such as HLA-E, HLA-F, and HLA-G in humans) tend to be less polymorphic, typically function in immune regulation, and exhibit more tissue-specific expression (LeMaoult et al. 2003).

Variation within MHC genes has frequently been linked with species’ susceptibility to disease, where higher levels of variation at MHC loci is thought to enhance a hosts ability to respond to a broader range of pathogen-derived antigens (Hughes and Yeager 1998; Penn et al. 2002). The association between MHC genes and disease resilience has been observed across multiple wildlife species including the northern leopard frog (*Rana pipiens*) (Trujillo et al., 2021), guignas (*Leopardus guigna*) (Napolitano et al. 2023) and desert bighorn sheep (*Ovis canadensis nelsoni*) (Dugovich et al. 2023), with disease-resilient populations exhibiting greater heterozygosity at MHC loci. The importance of the MHC in the immune cascade makes them excellent candidates to start to understand a populations’ adaptive genetic diversity. For many threatened species, the lack of a high-quality reference genome and high-quality immune gene annotations has limited our ability to investigate the repertoire and variation present within the MHC region (Peel et al. 2022). Research on the MHC region in non-avian reptiles has included studies on the komodo dragon (*Varanus komodoensis*) (Reed and Settlage 2021) and the tuatara (*Sphenodon punctatus*) (Gemmell et al. 2020; Miller et al. 2005). More recently, genomic analyses in two anole species (*Anolis carolinensis* and *Anolis sagrei*) (Card et al. 2022) and the Chinese alligator (*Alligator sinensis*) (He et al. 2022b), have provided a more comprehensive characterisation of reptile MHC.

**Fig. 1:**
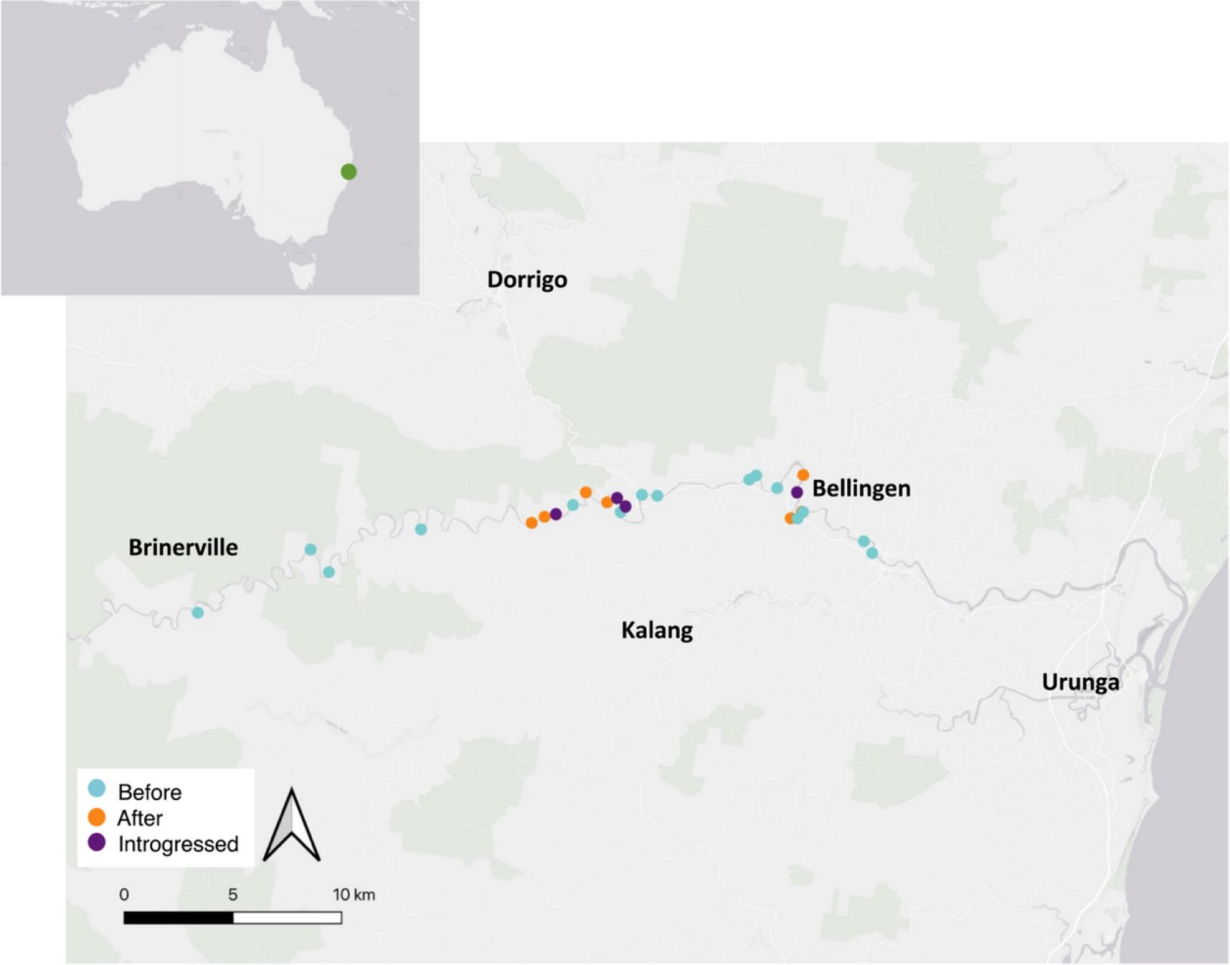
An inset of Australia with a map of the Bellinger River basin showing the locations of historic (before: 2007, blue), contemporary (after: 2019, orange), and backcross (introgressed: 2019, purple) samples collected across the species’ range (NSW Department of Climate Change, Energy, the Environment and Water (DCCEEW) (unpublished data)

The critically endangered Bellinger River turtle (*Myuchelys georgesi*) is a medium-sized omnivorous turtle that is restricted to a 60 km stretch of Bellinger River, on New South Wales mid-north coast (Fig. 1). The Bellinger catchment, along with several other freshwater catchments in NSW, remains isolated and largely undisturbed. Cut-off by the ocean to the east and the Great Dividing Range to the west, these geographic boundaries have promoted unique habitat specialisation and catchment-specific speciation (Spencer et al. 2014). The species consists of a single wild population making it highly susceptible to stochastic events. In recent years, the species is estimated to have undergone a population decline of over 90% due to nidovirus outbreaks of unknown origin (Chessman et al. 2020; Zhang et al. 2018).

In 2015, a species-specific nidovirus outbreak (the Bellinger River virus; BRV) caused the population to crash from *ca.* 4000 individuals to *ca.* 200 and resulted in a significant decline in genetic diversity, as measured by reduced representation sequence data (Chessman et al. 2020; Nelson et al. 2024). The near extinction of the species led to urgent conservation efforts including the development of a *M. georgesi* conservation action plan, a range of community engagement initiatives, habitat restoration projects, and the establishment of a conservation breeding program comprised of two populations founded from 16 and 19 wild individuals. Since 2015, two lesser-documented viral outbreaks have occurred in the river, one in January of 2022 (Parrish et al. 2024) and a second in May of 2024 (NSW DCCEEW, *pers. comm*., 2024). The ongoing disease outbreaks puts the species at high risk of extinction and requires investigation into potential genetic mechanisms that have led to this susceptibility.

Interestingly, one of the species’ closest relatives, the Murray River turtle (*Emydura macquarii*), demonstrates resilience to the viruses. *E. macquarii* is widespread along the east coast of Australia, and it is hypothesised that the species has been introduced to the Bellinger River via human-mediated dispersal over the past two decades (Spencer et al. 2018). *E. macquarii* is known to hybridise with *M. georgesi* (Georges et al. 2018). Pure *E. macquarii*, F1 hybrids (*M. georgesi* × *E. macquarii*), and wild-caught F2 backcrosses to *M. georgesi*, previously identified by Georges et al. (2018), have tested positive to the recent viruses, but do not exhibit symptoms or succumb to the disease (Zhang et al. 2018). Given the high susceptibility of *M. georgesi* to nidovirus infections, conservation managers are looking for long-term viable options without the need for continual intensive management. A common strategy to enhance genetic diversity and potentially mitigate disease susceptibility is the introduction of individuals from genetically diverse or distinct populations (Frankham 2015; Frankham et al. 2017). Since this is not feasible for *M. georgesi*, alternative strategies may be required to enhance genetic diversity for long-term population viability such as interspecific hybridisation (Baack and Rieseberg, 2007), or the reintroduction of historical genetic variation lost over time through methods such as back-breeding, cloning, or genome editing (Shapiro 2017).

Previous work has shown that this species exhibits low genome-wide diversity (Nelson et al. 2024), characterised by limited intra-population differentiation and low allelic richness compared to other testudines populations (Buchanan et al. 2019; Fay et al. 2023). These findings, derived from reduced representation sequencing (RRS) data across 460 putatively neutral markers (Nelson et al. 2024), likely reflect the species’ restricted geographic range, limited dispersal capacity, and long generation time. Additional analyses hypothesise that the species has undergone a number of historical bottlenecks, which have likely contributed to its low genetic diversity and ongoing disease susceptibility (Georges 2020).

In this study, we undertook manual MHC gene annotation by characterising the genetic features of MHC I and MHC II genes in *M. georgesi*. Building on previous research limited to 460 putatively neutral single nucleotide polymorphisms (SNPs), we use whole-genome re-sequencing (WGR) to analyse 12 contemporary turtles sampled after the disease outbreak, 19 historic turtles sampled before the outbreak, and four opportunistically sampled backcross individuals, to undertake higher-resolution, genomic investigations. Here we aimed to (1) assess the effect of deep historical events on genome wide-diversity relative to backcross animals and effective population size, and (2) assess the effect of a recent bottleneck in the past 15 years due to a viral outbreak on genome-wide diversity and diversity within the MHC genes by comparing the before and after outbreak groups.

## Methods

### Immune gene annotation

We used a homology-based approach via BLAST v2.3.30 (Camacho et al. 2009) to manually characterise all MHC class I and II genes in the *M. georgesi* genome generated by Nelson et al. (2024) (rMyuGeo1.pri.20230808; GCA_040894355.1). The genome is 2 Gb, consists of 128 scaffolds and has an N50 of 123.4 Mb (Nelson et al. 2024). The high contiguity of the genome suggests that the MHC region is likely well assembled, providing a strong foundation for reliable MHC gene characterisation and downstream analyses (Peel et al. 2022). To annotate class I genes we acquired query sequences from the National Centre for Biotechnology Information (NCBI) including the tawny dragon (*Ctenophorus decresii*) (KY905241.1), caiman (*Caiman crocodilus*) (KF769542.1), marine iguana (*Amblyrhynchus cristatus*) (EU839663.1), Galapagos land iguana (*Conolophus subcristatus*) (EU604313.1), tuatara (DQ145788.1), and green sea turtle (*Chelonia mydas*) (OK135213.1) (Table S1). To annotate class II, we acquired query sequences from NCBI including the Chinese softshell turtle (*Pelodiscus sinensis*) (MT834970.1), marine iguana (FJ623752.1), and green-rumped parrotlet (*Forpus passerines*) (EF710746.1) (Table S1). We used query sequences from multiple organisms for manual annotation to improve gene predictions by increasing the likelihood of matching conserved MHC elements. Query sequences were used to search the *M. georgesi* genome and a global transcriptome generated by Nelson et al. (2024), using BLASTn and tBLASTn, respectively, with an *e*-value threshold of 1e-10. Exon splicing sites were manually checked by visualising against the reference genome, global transcriptome, and automated annotation in IGV v.2.16.0 (Robinson et al. 2011). Nucleotide sequences for each gene were then extracted from the reference genome using BEDtools v2.29.2 (Quinlan and Hall 2010) and input into MEGA v11 (Tamura et al. 2021). Nucleotide sequences were converted to protein sequences to ensure no stop codons were present within exons. Pairwise nucleotide similarity between genes was calculated using EMBL-EBI Clustal Omega (Madeira et al. 2019). To classify MHC genes as classical or non-classical, we analysed gene expression by aligning transcripts from brain, liver, and spleen tissues to MHC genes using IGV v.2.16.0. Phylogenetic relationships between genes within each class were inferred using sequences acquired from a maximum likelihood (ML) analysis performed in IQ- TREE2 (Minh et al. 2020). When possible, complete coding sequences for reptiles and amphibians were acquired from NCBI; however, predicted sequences were used for the inclusion of testudines data (Table S2, Table S3). The ModelFinder option (-m MFP) within IQTREE2 was used to select the best-fitting substitution model according to the Bayesian information criterion (Kalyaanamoorthy et al. 2017). Node support was assessed using the ultrafast bootstrap (-bb 1000) approximation and the like approximate likelihood-ratio test (-alrt 1000) (Guindon et al. 2010; Hoang et al. 2018).

### Re-sequenced genome sampling, extraction, and sequencing

Re-sequenced genomes were generated using samples collected by the NSW Department of Climate Change, Energy, the Environment, and Water (DCCEEW) during surveys before the disease outbreak in April of 2007, after the outbreak in November 2019, and from F2 backcrosses to *M. georgesi* in November 2019 (Table S4). DNA samples were collected by removing part of the trailing webbing of the clawless toe on the hindfoot or by extracting blood from the jugular vein (Georges et al. 2018) (Table S4). Samples were stored in 75% ethanol at − 20 °C in the University of Canberra Wildlife Tissue Collection (GenBank UC < Aus >). For DNA extractions we performed a high salt method on 19 skin tissue biopsies and 16 dried bloods on Whatman card samples following a modified protocol from Aljanabi and Martinez (1997) that included an additional round of spin column centrifugation. DNA concentration and quality were assessed using a Nanodrop 2000 Spectrophotometer (ThermoFisher Scientific) and 0.95% agarose gel electrophoresis for 30 min at 90 V. To maximise DNA quality for WGR, we undertook an additional DNA repair step using a FFPE DNA repair protocol (New England Biosciences). Repaired DNA concentration and quality were assessed using a Nanodrop 2000 Spectrophotometer (ThermoFisher Scientific) and 0.95% agarose gel electrophoresis for 30 min at 90 V. Samples were sent to Ramaciotti Centre for Genomics (University of New South Wales, Australia) for WGR. To minimise batch effects, all samples were processed simultaneously on an Illumina NovaSeq 6000, using a TruSeq DNA PCR free library prep kit across six lanes.

### Re-sequenced genome alignment

Raw 150 bp paired-end FASTQ reads from 35 individuals were quality checked and trimmed using fastQC v0.11.8 (Andrews 2010) and trimmomatic v0.39 (Bolger and Giorgi 2014) with the parameters ILLUMINACLIP:TruSeq3-PE.fa:2:30:10 SLIDINGWINDOW:4:5 LEADING:5 TRAILING:5 MINLEN:25. Reads were aligned to the reference genome using Burrows-Wheeler aligner (BWA) v0.7.17 (Li and Durbin 2009) ‘mem’ function with default parameters. The resulting alignment files were sorted into BAM format using SAMtools (Li et al. 2009) sort v1.6 and alignment rates and coverage calculated using SAMtools v1.6 ‘flagstat’ and ‘depth’, respectively (Li et al. 2009) (Table S4). As individuals were sequenced across multiple lanes, BAM files pertaining to a single individual were merged using SAMtools merge and duplicates marked and removed using picard v2.21.9 MarkDuplicates (http://broadinstitute.github.io/picard/). For genome-wide investigations, we partitioned our data into three putative groups (two temporal, reflective of groups investigated by Nelson et al. (2024)); wild individuals sampled before the disease outbreak in 2007, *N* = 19 (hereafter ‘before’); wild individuals sampled after the disease outbreak between 2015–2020, *N* = 12 (hereafter ‘after’); and F2 backcrosses to *M. georgesi*, *N* = 4 (hereafter ‘backcross’). For demographic history and MHC characterisation, the backcross group was excluded to avoid confounding effects on historical demography. Additionally, within the scope of this study, the absence of *E. macquarii* annotations, combined with the limitations of short-read data, posed a risk to the reliability of accurate variant calls (Figure S1).

### Genome-wide diversity

To investigate genome-wide diversity, autosomal heterozygosity and runs of homozygosity (ROHs) were calculated across each genome (*N* = 35) using ROHan (Renaud et al. 2019). ROHan combines a local Bayesian model and hidden Markov model (HMM) to identify autosomal heterozygosity and ROHs from BAM files of individually mapped genomes (Renaud et al. 2019). Analyses were run on nine macrochromosomes, excluding the putative sex chromosome (scaffold 4) which was identified through genome coverage analyses (Nelson, unpublished data) and inferred from the *E. macquarii* karyotype (Martinez et al. 2008). We ran ROHan with three parameters; –rohmu 5e-5, –TsTv 1.965, -t 4 and –size 100,000 (100 kb windows) on an Amazon Web Services ubuntu 20.04 LTS cloud machine (r5.8 × large, 32 vCPU, 256 Gb RAM, 1 TB attached storage). Inbreeding coefficient based on ROH (F_ROH_) (McQuillan et al. 2008) and average individual-level heterozygosity was calculated for each group using the hmmrohl and summary texts output by ROHan, respectively. Results were visualised in R v4.3.0 using dplyr() v1.1.2. and ggplot2() v3.5.0. We also calculated the number of genome-wide variants using BCFtools v1.3.1 ‘call’ (Danecek et al. 2021).

### Demographic reconstructions

Changes in ancient effective population size (*N*_*e*_) over time were assessed with the pairwise sequentially Markovian coalescent (PSMC) model (Li and Durbin 2011) using sorted BAM files for pure *M. georgesi* (*N* = 31). For demographic reconstructions, sites with coverage below one-third or above twice the sample’s average coverage were removed following the approach of Bi et al. (2020). Additionally, we included only scaffolds > 50 kb and excluded the sex chromosome. Individual consensus genome sequences containing SNP variants were generated for 31 M*. georgesi* using SAMtools v1.6 with the ‘mpileup’ command. ‘mpileup’ was used to directly represent sequencing variation without additional filtering, minimising potential biases in demographic inference (Li et al. 2009). For PSMC analyses, the files were processed using ‘vcfutils.pl’ and ‘vcf2fq’ to call SNP variants, following the recommended protocol (https://github.com/lh3/psmc). PSMC v0.6.5-r67 was run with the following default parameters, -N25 -t15 -r5 -p ‘4 + 25 × 2 + 4 + 6’. Results were scaled by a mutation rate of 4.61 × 10^−9^ substitutions per site per generation based on estimates for *Chrysemys picta *(Bergeron et al. 2023). As precise details of the life history of *M. georgesi* are not available, we used a generation time of 14 years based on manager recommendations. To investigate recent historical patterns of demography we calculated *N*_*e*_ using GoNe (Santiago et al. 2020). Following the SAMtools v1.6 ‘mpileup’ step mentioned above, we used BCFtools ‘call’ for multi-sample variant calling. For stringent filtering, we retained variants with a MAF of > 0.04, where the alternate allele must be seen twice in the population, which could be homozygous in a single individual or heterozygous in two individuals. As a result, 2,979 variants were removed from the total of 3,681,055 (Table S8), as they were more likely to represent genome-wide sequencing errors than true variants in this genetically homogeneous species. We conducted multiple iterations of GoNe with default parameters to determine the optimal values for *hc* (0.01, 0.05, 0.1) and various recombination rate estimates (1, 2, 3). The optimal values were identified as a recombination rate of 3 cM/Mb and an *hc* of 0.01. *N*_*e*_ outputs were visualised using ggplot2() v3.5.0 in R v4.3.0.

### MHC gene variant calling and analyses

To characterise MHC diversity in the before and after samples (*N* = 31), we generated a joint-genotyped dataset. This was done using GATK GenomicsDBImport to create a sample database, followed by GATK GenotypeGVCF to produce a whole-genome joint-genotyped multi-sample VCF file containing both temporal groups. As previously mentioned, backcross animals were excluded from these analyses due to the absence of manually annotated MHC genes in *E. macquarii* and concerns about the reliability and confidence of short-read alignment and variant calls in the MHC core region. This is because uncharacterised gene duplication and structural factors can introduce artifacts when interspecific short reads are mapped to the genome (Jaegle et al. 2023) (Table S4), resulting incomplete or inaccurate variant identification and interpretation. We filtered the joint genotyped multi sample VCF using VCFtools v0.1.14 (Danecek et al. 2011), retaining SNPs, multiple nucleotide polymorphisms (MNPs), non-biallelic SNPs, and indels, to obtain raw variant counts within our manually annotated MHC exons using BCFtools (Table 2). Among these variant types, SNPs were the most abundant, providing the highest level of variation and making them the most informative for downstream analyses. For SNP analyses, we filtered the VCF to include only biallelic SNPs found within the MHC exons. We applied GATK VariantFiltration and SelectVariants v4.2.0.0 (McKenna et al. 2010) using GATK-recommended thresholds to remove variants with MQ < 40, − 12.5 < MQRankSum < 12.5, − 8 < ReadPosRankSum < 8, and a stringent QUAL score of < 80. BCFtools was then used to filter out sites with an average depth across all samples of < 10 and those with an allelic balance > 0.9. Finally, we used VCFtools to retain SNPs with a minor allele frequency (MAF) > 0.01, to retain rare variants within the MHC region. Using the filtered biallelic SNP set, we then calculated the number of SNPs in each gene and determined if they resulted in synonymous or non-synonymous amino acid substitutions using Geneious Prime 2020 (https://www.geneious.com/). To identify full length allele sequences for each MHC gene in each individual, we undertook phasing. First, we used GATK FastaAlternateReferenceMaker (v4.2.0.0) (McKenna et al. 2010) to generate single sample FASTA sequences for each gene. SeqPHASE (Flot 2010) was used to convert the FASTA sequences into phase format then PHASE (v.2.1.1) (Stephens et al. 2001; Stephens and Scheet 2005) was run to generate alleles and SeqPHASE used to convert phase output into phases FASTA sequences to give 70 sequences for each gene (two per individual). We calculated observed (*H*_o_) and expected heterozygosity (*H*_E_) across the complete MHC I and MHC II exon sequences in temporal (before and after) using GenAlEx v6.5 (Peakall and Smouse 2006) and visualised average individual *H*_o_ by group using the boxplot() functions in R. To assess genetic differentiation among individuals, we generated principal component analysis (PCoA) plots using our genome-wide and MHC joint genotyped VCFs, using the adegenet package (Jombart 2008) in R (v4.1.1) (R Core Team 2023). Comprehensive details of sample metadata and a breakdown of the methodological approaches used in this study are shown in Table S4 and Figure S1, respectively.

## Results

### MHC annotation

**Fig. 2:**
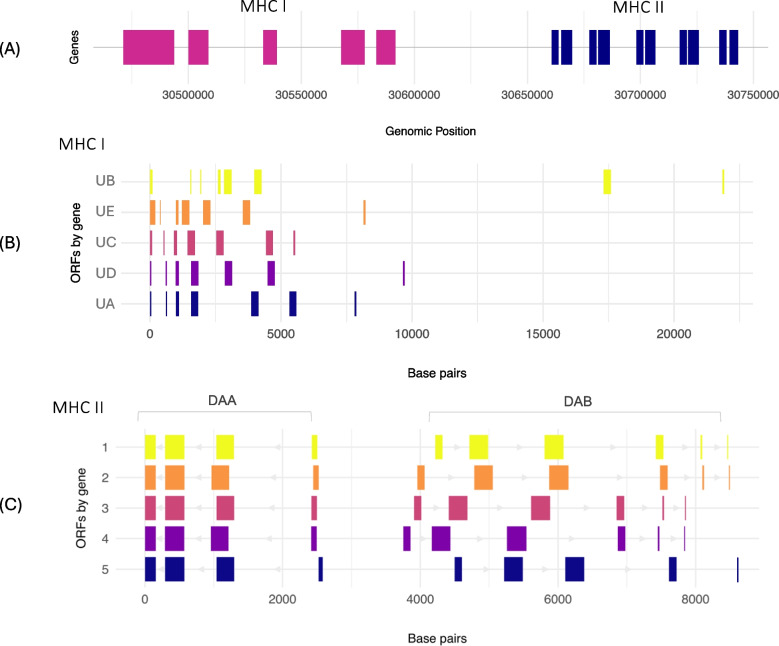
Genomic architecture of major histocompatibility complex (MHC) genes. **A** Genomic architecture of the core major histocompatibility complex (MHC) region, including class I and class II genes, on scaffold 10. **B** and **C** Genomic coordinates and structural organisation of manually annotated *Myuchelys georgesi* (*Myge*) MHC genes presented as open reading frames (ORFs), in order of genomic position along scaffold 10

Through manual annotation of immune-related genes, we identified five MHC class I loci (*Myge-UA*, *Myge-UB*, *Myge-UC*, *Myge-UD*, *Myge-UE;* labelled from most to least number of detected variants), and ten MHC class II loci (*Myge-DAA1*, *Myge-DAB1*, *Myge-DAA2*, *Myge-DAB2*, *Myge-DAA3*, *Myge-DAB3*, *Myge-DAA4*, *Myge-DAB4*, *Myge-DAA5*, *Myge-DAB5*), all located within a 272,213 bp region on scaffold 10. The gene nomenclature adheres to the conventions established by Miller et al. (2005) and Miller et al. (2015) for a reptilian species; however, limited annotations and published sequences in other reptile species made comparative interpretation challenging. The *M. georgesi* MHC class II genes are densely clustered, consisting of alpha (here labelled *DAA*) and beta (here labelled *DAB*) chains encoded on the 3′ to 5′ and 5′ to 3′ strands, respectively (Table 1). Structurally, the class II genes exhibit high conservation, except for *Myge-DAB5* which shows a putative loss of exon 5 with no transcriptional evidence across three tissue types (Fig. 2). Similarly, MHC class I genes display a largely conserved architecture, except for *Myge-UA*, which has notably larger introns 1 and 2 compared to other MHC class I genes and contains an additional exon (Fig. 2; Table S5). Differentiation analyses revealed high sequence similarity between *Myge-UB* and *Myge-UE*, whereas *Myge-UD*, *Myge-UA*, and *Myge-UC* exhibited closer sequence similarity (Table S6 A). As expected, lower levels of differentiation were observed within the MHC II alpha (*DAA*) and beta (*DAB*) gene groups compared to between these gene groups (Table S6B). Transcriptome data did not provide a clear distinction between classical and non-classical Class I genes, as transcript levels across the three tissue types (brain, liver, and spleen) were consistent for all genes, suggesting that additional tissue types may be needed to identify if any of the identified class I genes may have non-classical functions.

**Table 1:** Manually annotated MHC I and MHC II genes on scaffold 10 used for downstream analyses. Start and end coordinates refer to whole-genome coordinates on the reference genome (GCA_040894355.1). Coordinates for each exon are presented in table S5

Class	Gene	Scaffold	Exons	Start	End	Strand
**MHC I**	*Myge_UB*	10	8	30,471,574	30,493,484	-
	*Myge_UE*	10	7	30,500,310	30,508,525	-
	*Myge_UC*	10	7	30,533,405	30,538,940	-
	*Myge_UD*	10	7	30,567,953	30,577,665	-
	*Myge_UA*	10	7	30,583,497	30,591,368	-
**MHC II**	*Myge_DAA1*	10	4	30,660,982	30,663,487	-
	*Myge_DAB1*	10	6	30,665,109	30,669,452	+
	*Myge_DAA2*	10	4	30,677,698	30,680,223	-
	*Myge_DAB2*	10	6	30,681,654	30,686,193	+
	*Myge_DAA3*	10	4	30,698,489	30,700,988	-
	*Myge_DAB3*	10	6	30,702,397	30,706,348	+
	*Myge_DAA4*	10	4	30,717,695	30,720,191	-
	*Myge_DAB4*	10	6	30,721,446	30,725,542	+
	*Myge_DAA5*	10	4	30,735,202	30,737,782	-
	*Myge_DAB5*	10	5	30,739,701	30,743,969	+

To contextualise the genetic relationships among MHC homologs in *M. georgesi* and extend these comparisons across non-avian and avian reptiles, we constructed phylogenetic trees using complete coding sequences of MHC I and MHC IIB sequences. The resulting phylogenies showed that *M. georgesi* forms monophyletic groups with other testudines across both classes, with certain *M. georgesi* genes forming unique clades alongside other testudines (*Myge*-UA, *Myge*-UB, *Myge*-UE, *Myge*-DAB4) (Figure S2; Figure S3). Furthermore, testudines and members of the Crocodilia were shown to form a monophyletic group, separate from Squamata, Aves, and Rhynchocephalia in the MHC class II tree (Figure S3).

### MHC diversity

Following the identification and filtering of MHC variants, we identified 257 biallelic SNPs across all MHC exons in the before group, including 127 SNPs in class I and 136 SNPs in class II genes (Table 2). In the after group, 232 biallelic SNPs were identified across all MHC exons, comprising 108 SNPs in class I and 124 SNPs in class II genes (Table 2). The SNPs were unevenly distributed among MHC genes, with highest number of variants contained in *Myge-UA* (*N* = 66) and the lowest number of variants contained in *Myge-DAB3* which was monomorphic (Table 2). Overall, the 14 genes with variants had an average of 18 exonic SNPs (range: 3–66), most of which were in exons 2, 3, and 4 across both classes. Among the 257 SNPs identified in the ‘before’ animals, 171 (67%) were predicted to result in non-synonymous substitutions. In the ‘after’ animals, 154 of the 232 (59%) detected variants were also predicted to be non-synonymous. Non-synonymous variation was observed across 14 genes in the ‘before’ group and 13 genes in the ‘after’ group, respectively. A total of 15 and 13 alleles were observed in MHC I and 35 and 27 alleles were observed in MHC II in before and after groups, respectively (Table 2). The before population had one private allele in *Myge-UC* and *Myge-UE,* and 11 private alleles across *Myge-DAA1*, *Myge-DAA2, Myge-DAB4, Myge-DAA5, Myge-DAB5* that were unique to that population. We found no significant differences in allelic diversity between temporal groups (MHC I: *t* = 1.8711, df = 8, *p*-value = 0.09823; MHC II: *t* = 1.688, df = 16, *p*-value = 0.1108) (Table 2). On average, class I genes had higher numbers of SNPs, non-synonymous SNPs, and fewer alleles compared to class II. SNP *H*_O_ and *H*_E_ was similar across temporal groups (Table S9) with slightly lower observed than expected heterozygosity at MHC loci. Higher number of variants corresponded with higher levels of heterozygosity across both classes in pure *M. georgesi*. PCoA analyses revealed no clear temporal differentiation in MHC genes, although clustering of individuals was observed, potentially reflecting the presence of shared alleles (Figure S6). Slightly greater differentiation was observed in the ‘before’ group for MHC I genes (Figure S6 A), indicating a minor degree of temporal structuring. In contrast, genome-wide analyses showed no discernible clustering patterns. The inclusion of backcross individuals resulted in tighter clustering of purebred animals, reflecting the nearly identical genome-wide diversity within the species and the absence of distinct genetic structuring (Figure S7 A). This observation was further validated by the exclusion of backcross individuals, which revealed no significant clustering or differentiation among pure groups, suggesting minimal genetic divergence over time (Figure S7B).

**Table 2:** Temporal variant statistics for each MHC gene investigated including number of indels; non-biallelic single nucleotide polymorphisms (SNPs); multiple nucleotide polymorphisms (MNPs); filtered SNPS; non-synonymous SNPs (ns); ratio of synonymous to non-synonymous SNPs (dN/dS); number of alleles; and allelic diversity. Before *N* = 19, after *N* = 12. Zero denoted by ‘-’

			Before								After							
Class	Gene (*Myge*)	ORF length (bp)	Indels	Non-biallelic SNPs	MNPs	SNPs	SNPs (ns)	SNP dN/dS	No. alleles	Allelic diversity	Indels	Non- biallelic	MNPs	SNPs	SNPs (ns)	SNP dN/dS	No. alleles	Allelic diversity
**MHC I**	*UA*	1063	-	-	-	66	40	0.606	4	0.610	-	-	-	60	40	0.606	3	0.513
	*UB*	1124	4	-	-	23	16	0.696	3	0.4–92	4	-	-	23	16	0.696	3	0.444
	*UC*	1096	-	-	-	18	15	0.833	4	0.495	-	-	-	12	9	0.750	3	0.513
	*UD*	1096	-	-	-	8	6	0.750	2	0.499	-	-	-	8	6	0.750	2	0.555
	*UE*	1196	1	2	-	6	1	0.167	2	0.512	1	2	-	5	-	0.000	2	0.555
**MHC II**	*DAA1*	774	-	-	1	16	13	0.813	7	0.697	-	-	1	13	9	0.563	5	0.506
	*DAB1*	805	-	-	1	12	1	0.083	2	0.493	-	-	1	12	1	0.083	2	0.444
	*DAA2*	774	-	-	-	3	3	1.000	3	0.370	-	-	-	3	3	1.000	2	0.180
	*DAB2*	807	-	-	-	16	12	0.750	3	0.553	-	-	-	11	9	0.563	3	0.537
	*DAA3*	774	-	-	-	4	4	1.000	2	0.347	-	-	-	4	4	1.000	2	0.297
	*DAB3*	804	-	-	-	-	N/A	N/A	N/A	N/A	-	-	-	-	N/A	N/A	N/A	N/A
	*DAA4*	775	-	-	-	29	22	0.759	3	0.590	-	-	-	27	21	0.724	3	0.509
	*DAB4*	807	-	-	-	17	13	0.765	5	0.674	-	-	-	15	11	0.647	4	0.675
	*DAA5*	751	-	-	-	27	17	0.630	7	0.763	-	-	-	27	17	0.630	4	0.555
	*DAB5*	777	1	-	-	12	8	0.667	3	0.562	1	-	-	12	8	0.667	2	0.486

### Genome-wide diversity and demographic history

**Fig. 3:**
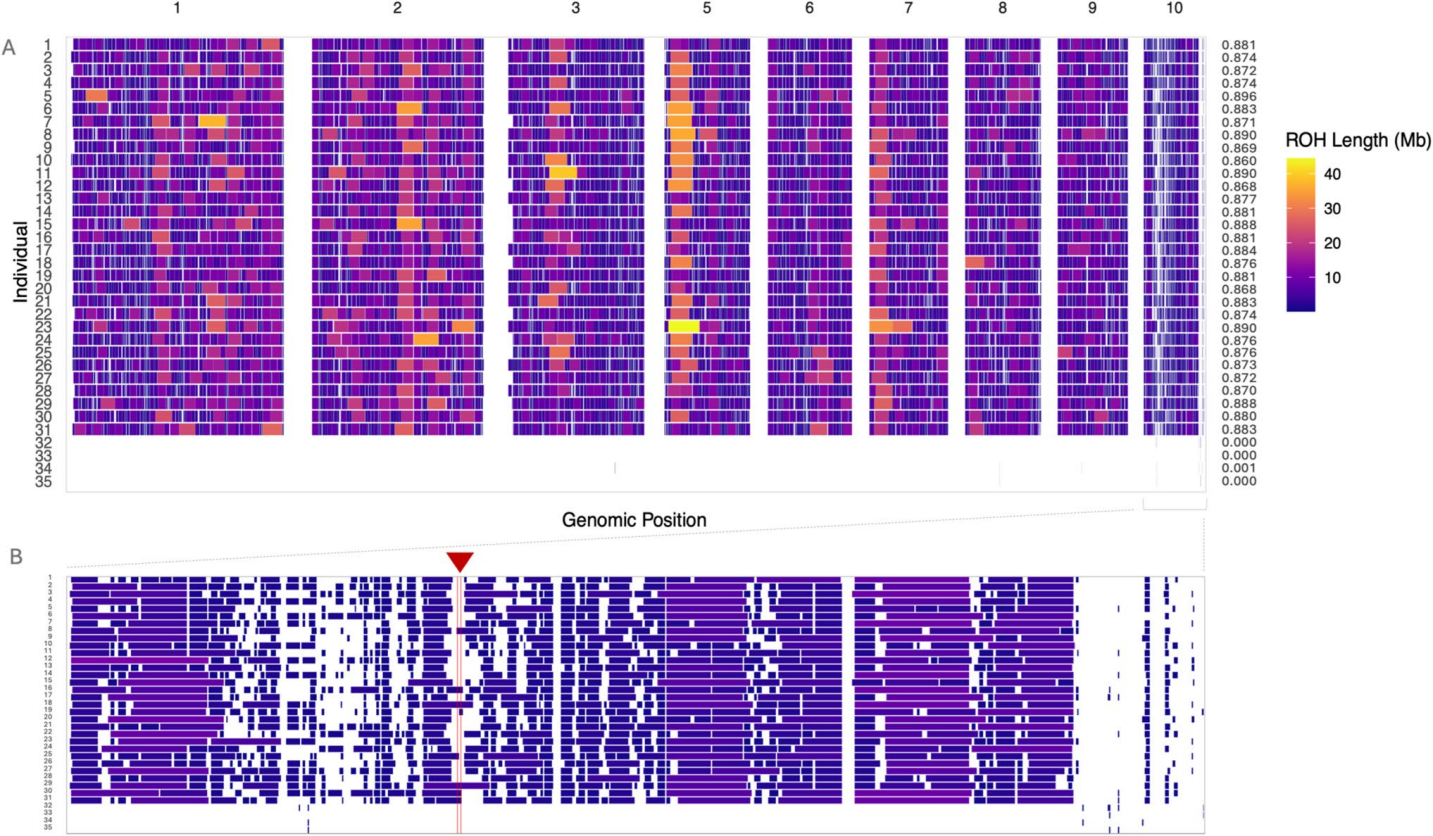
Runs of homozygosity (ROH) heatmap illustrating **A** the chromosomal distribution, length, and the proportion of the genome in ROH across nine macrochromosomes in before (individuals 1–19) and after (individuals 20–31), relative to F2 backcrosses (individuals 32–35). **B** Enlarged view of scaffold 10. Major histocompatibility complex (MHC) core region marked by a red arrow and parallel lines

All whole-genome samples had an average alignment rate of 99.6% and 99.5% for pure and backcross animals, respectively, across ten putative macrochromosomes (scaffolds 1–10). Raw SNP calls across the genomes were substantially higher in backcross animals (~ 15,000,000) compared to pure *M. georgesi* (~ 650,000) (Table S7). ROH were distributed across all macrochromosomes (Fig. 3A). The longest Run of Homozygosity (ROH) was identified on scaffold 5 in purebred individuals and covered 36% of the scaffold’s total length (Fig. 3A). In contrast, scaffold 10 exhibited the lowest density of ROH relative to its size (Fig. 3A). The pure samples had 90% of their genome in ROH, characterised by short (< 2 Mb) and long (> 2 Mb) ROH as defined by Ceballos et al. (2018), while only a small number of short ROH were detected in backcross individuals (Fig. 3A, Table S7, Figure S4, Figure S5). Small ROHs measuring 0–500 kb, 500–1000 kb, and 1000–2000 kb had similar frequencies across groups (Figure S5 A). Although counts of small and long ROH were relatively similar, the proportion of the genome in long runs was notably higher than small runs (Figure S5B). This is consistent with the expectation that larger ROHs occupy a greater proportion of the genome. The before and after samples had an identical proportion of the genome in ROH and comparable ROH lengths. The average number of ROH per individual was also consistent between the two temporal groups. No significant differences in nucleotide diversity across the genome were observed between the before and after individuals, regardless of whether ROH were included or excluded. Both temporal groups displayed significantly lower nucleotide diversity compared to the backcross individuals (Table S7).

**Fig. 4:**
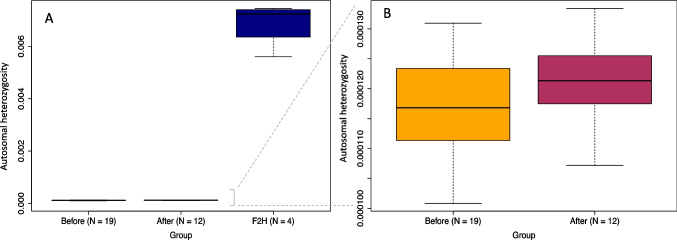
Genome-wide heterozygosity estimates for *Myuchelys georgesi* calculated using ROHan. Boxes represent the interquartile range (IQR) with whiskers extending to the upper and lower ranges and the bold line representing the group mean. **A** Autosomal heterozygosity estimates for all groups including before, after, and backcross. **B** Rescaled autosomal heterozygosity estimates of pure *M. georgesi* before and after groups plotted in A

We identified significantly lower levels of genome-wide SNPs (Table S7) and autosomal heterozygosity in pure versus backcross animals (pure: 1.18 × 10^−4^; backcross: 6.8 × 10^−3^, *t* = − 16.01, *p* = 0.001) (Fig. 4A). No significant difference was observed between temporal groups (before: 1.17 × 10^−4^; after: 1.21 × 10^−4^, *t* = − 1.40, *p* = 0.1745) (Fig. 4B).

**Fig. 5:**
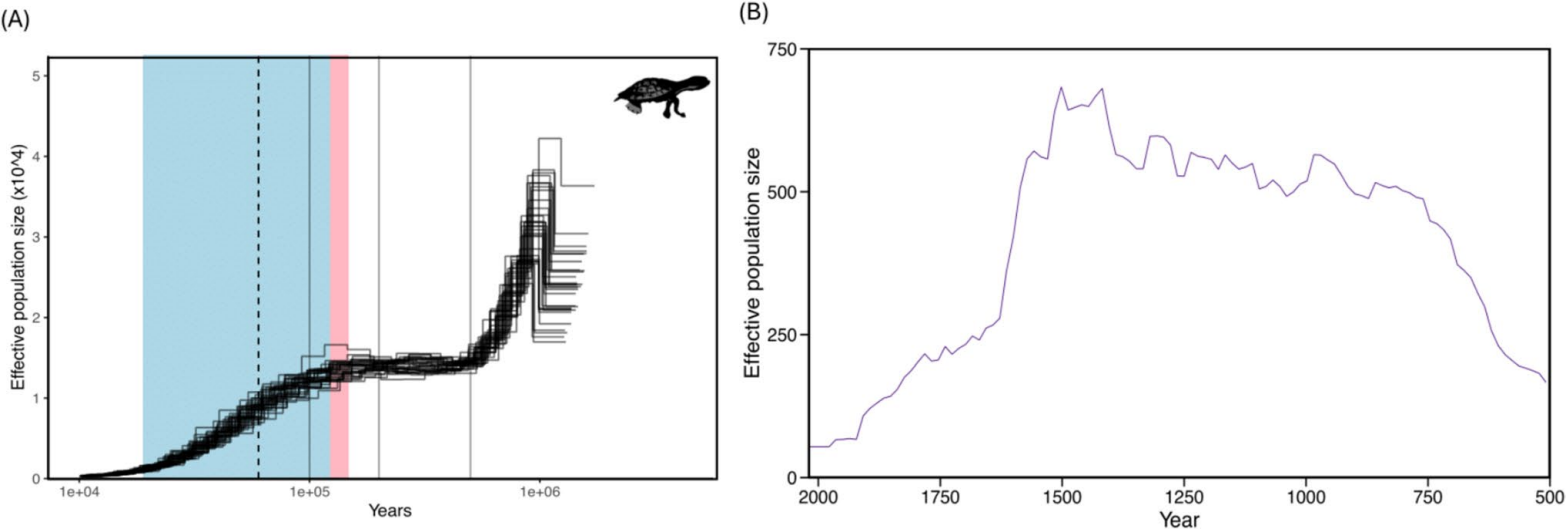
Changes in effective population size (*N*_*e*_) over time estimated using **A** PSMC and **B** GoNe for *Myuchelys georgesi*. The x axes indicate time before present in years, and the y axis indicates the effective population size. A generation time of 14 years was used for both analyses. For PSMC analyses, axes were scaled by mutation rate of 4.61 × 10^−9^ substitutions per site per generation. PSMC rectangles correspond to the last inter-glacial (red, warm) and glacial period (blue, cold). Vertical dotted line represents arrival of humans to Australia

Our demographic history reconstructions using PSMC analysis indicate a gradual, long-term decline in effective population size beginning approximately 110,000 years ago, coinciding with the last glacial period (Fig. 5A). The largest effective population size estimates correspond to a period before the last interglacial, around 110,000 years ago. These estimates were consistent across all 31 individuals analysed. Estimates predating this timeframe are likely less reliable due to including the accumulation of errors in coalescent events in deep time and limited resolution in ancient periods; therefore, they should be approached with caution. GoNe analyses showed there has been a decline in effective population size from an estimate *N*_e_ of 700, 50 generations ago to 100 in the current generation (Fig. 5B).

## Discussion

Here we provide a critical first step towards understanding the genome-wide and immune gene diversity in the Bellinger River turtle. We found low levels of genome-wide diversity in pure *M. georgesi* compared to backcross animals and evidence of long-term, continual declines in effective population size. We present the first comprehensive overview of the location and structure of the MHC region in a freshwater turtle species. Based on our manual annotations of the MHC genes, we observed relatively low levels of genetic variation within this gene region in pure *M. georgesi* relative to disease-resilient hybrids. However, scaffold 10, which encompasses the MHC region, exhibited higher levels of variation compared to other macrochromosomes. Consistent across both genome-wide and immune gene analyses, no significant changes in genetic diversity were detected before and after the disease outbreak.

Leveraging a scaffold-level genome assembly and RNAseq data, we identified MHC genes localised within a single core region, consistent with observations in many amniote species (Fig. 2A). Like *M. georgesi,* these studies revealed a single-core MHC region with linked class I and class II subregions. While the relocation of MHC genes to other genomic regions has been observed in reptiles—for instance, the displacement of class I and class II genes from the core MHC region in tuatara and anole (Card et al. 2022; Gemmell et al. 2020; Miller et al. 2015) —we did not find evidence for extensive duplication of functional MHC genes outside of the core region, aside from incomplete pseudogenes lacking major exons. This suggests a highly conserved MHC region with potential constraints on duplication to other regions of the genome in *M. georgesi*. The genomic organisation of each class forms two distinct clusters observed within the single-core MHC region as observed in the Chinese alligator (He et al. 2022b) and komodo dragon (Reed and Settlage 2021), with no interspersal of classes as observed in the tuatara (Gemmell et al. 2020), and some amphibian and mammalian species (He et al. 2023; Peel et al. 2019). These topologies align with the known evolutionary relationships within Reptilia (Zardoya and Meyer 2001), supporting both the conservation of MHC loci and their divergence along lineage-specific trajectories. Phylogenetic analyses of immune genes similarly reflect these established relationships, with both MHC classes showing the closest divergence from Crocodilia (Figure S2, Figure S3). This raises the question of whether these clades represent classical and non-classical genes or suggest specialised roles within the *M. georgesi* immune system. In humans, for instance, classical HLA (human leukocyte antigen) class Ia genes, such as HLA-A, are involved in antigen presentation to immune cells, are highly polymorphic, and play a critical role in adaptive immunity (Le Bouteiller and Lenfant 1996). In contrast, non-classical HLA class Ib genes such as HLA-G and HLA-E exhibit specialised immunomodulatory functions, are highly conserved and characterised by limited polymorphism, and are predominantly expressed in immunologically significant tissues (LeMaoult et al. 2003). Consistent with the genomic architecture observed in amphibians, MHC class I introns in *M. georgesi* are notably longer than those of MHC class II, where MHC II DAA and MHC II DAB are tightly linked (He et al. 2023). These keys features suggest that the MHC region in *M. georgesi* is relatively simple as the core MHC I and MHC II regions tightly clustered and adjacent in the genome. This simplified genomic arrangement appears to have been largely conserved throughout the evolutionary history of tetrapods, including birds (He et al. 2022a), reptiles (Card et al. 2022; He et al. 2022b; Miller et al. 2015), and mammals (Silver et al. 2024), particularly in MHC II genes.

Our demographic reconstructions indicate that the effective population size of *M. georgesi* has been declining since the last interglacial period (Fig. 5), potentially due to limited gene flow and accumulation of runs of homozygosity. Mitochondrial data estimate that *M. georgesi* diverged from its close relative, *E. macquarii*, approximately 6.1 million years ago during a period of aridification (Le et al. 2013). Geographic isolation and habitat fragmentation likely contributed to the demographic trends identified in our PSMC analyses. The low *N*_e_ estimates (< 500) from approximately 10,000 years ago (Fig. 5A) suggest that climatic changes during the last glacial period may have influenced the genetic structure of *M. georgesi,* similar to patterns observed in other freshwater turtle species (Hilgers et al. 2024). However, unlike these common turtle species, which eventually reach an equilibrium in *N*_e_ (Hilgers et al. 2024), *M. georgesi* has continued to experience a gradual, long-term decline. The glacial period likely reduced habitat availability and fragmented freshwater populations, leading to isolation, speciation, and long-term declines in effective population size. The species isolation, combined with life-history traits, have likely intensified the impact of accumulated inbreeding, resulting in reduced fitness and an elevated risk of extinction over time (Frankham et al. 2017). The long-term isolation and gradual decline in effective population size represent key findings for the species, likely exacerbating the critically low levels of contemporary genome-wide diversity observed in heterozygosity and ROH analyses. Consequently, this accumulation of inbreeding effects, compounded by geographic barriers to dispersal, may have driven the species into an extinction vortex well before the onset of the Anthropocene. This is supported by the large proportion of the genome found in ROH (Fig. 3A), as long-term inbreeding and gene flow can directly influence ROH abundance (Ceballos et al. 2018; Foote et al. 2021; Hewett et al. 2023; Mooney et al. 2021). Short ROH are indicative of background relatedness or inbreeding resulting from distant common ancestry, while long ROH reflect recent parental relatedness or occur in genomic regions with low recombination rates (McQuillan et al. 2008; Pemberton et al. 2012). In *M. georgesi*, the ROH distribution includes both short (< 2 Mb) and long (> 2 Mb) segments (Fig. 3A, Figure S4, Figure S5). The elevated levels of homozygosity observed in the contemporary population are likely the consequence of both historical inbreeding among distant ancestors and recent background relatedness. This is further supported by the recent declines identified through GoNe analyses, which have likely contributed to the significant proportion of the genome comprising of long ROH (Fig. 5B, Figure S5B, Table S5) (Kardos et al. 2021; Pemberton et al. 2012). ROHs, which are typically regions identical by descent, are likely absent in backcross animals due to increased genetic diversity and reduced inbreeding in parental *E. macquarii* in addition to heterozygote advantage and levels of genetic divergence (7.8%) between the two species (Fielder et al. 2012). The absence of significant differences in both the number and length of ROH and in autosomal heterozygosity across temporal groups suggests that genomic signatures associated with the recent disease outbreak are not yet detectable at the genome-wide level in a species with long generation times. These findings conflict with previous analyses using 460 SNPs and a sample set of before = 92 and after = 38 (Nelson et al. 2024). As WGR leverages a dense array of markers spread across an entire genome, compared to the RRS data that surveyed 0.000023% of the genome, the increased statistical power and even genomic coverage of the genome-wide dataset yields results that are more robust and reliable (Jeon et al. 2024). Although these signatures are not apparent in the genome-wide data yet, these signatures may emerge in subsequent generations, potentially increasing the species’ trajectory towards extinction, emphasising the importance of ongoing genetic monitoring.

Interestingly, the terminal region of scaffold 10 shows a notable absence of ROH, with only a few ROH observed in certain individuals, including backcrosses (Fig. 3). This pattern could potentially result from various factors, such as telomeric recombination disrupting long ROH tracts at the chromosomal ends (Bosse et al. 2012), or the presence of a functionally important gene region as observed in our characterisation of the MHC core region. However, further investigation would be necessary to confirm this.

Here we found higher levels of expected (*H*_E_) than observed (*H*_O_) at MHC loci. We previously found higher *H*_O_ than *H*_E_ heterozygosity in the species using 460 putatively neutral SNP markers (Nelson et al. 2024). Although neutral genetic variation is frequently employed as a proxy for adaptive potential, our findings support the argument that neutral diversity may not fully capture adaptive potential, particularly when only a small set of RRS variants is considered. Instead, genetic diversity at specific functional loci provides a more accurate measure of a population’s adaptive potential (Holderegger et al. 2006; Teixeira and Huber 2021). MHC variants play a critical role in a wide range of biological traits. For instance (i) immune recognition where a single amino acid change in the antigen-binding region of the DRB*1302 allele in humans abolishes malaria recognition (Frank 2002; Summers et al. 2003); (ii) susceptibility to infectious and autoimmune diseases, as observed in MHC IIB heterozygotes, which exhibit lower Ranavirus infection intensity compared to homozygotes in larval wood frogs (*Rana sylvatica*) (Savage et al. 2019); (iii) individual odours and mating preferences seen in song sparrows (*Melospiza melodia*) (Grieves et al. 2019) and other aves (Leclaire et al. 2014), where preen oil odour is used to discern MHC similarity and diversity of potential kin or mates and; (iv) pregnancy outcomes observed in giant pandas (*Ailuropoda melanoleuca*), where mating pairs with MHC dissimilarity exhibit higher reproductive success (Sommer 2005; Zhu et al. 2019). High levels of inbreeding often lead to an overall decrease in MHC variants, loss of rare and potentially advantageous alleles, and a decreased ability to adapt to novel or rapidly evolving pathogens (Altizer et al., 2003; Spielman et al., 2004). Despite the low genome-wide diversity observed in *M. georgesi*, MHC genes appear relatively conserved. The lack of ROH seen in the core MHC region, and scaffold 10 in general, relative to other macrochromosomes suggests that SNP diversity alone, may not be a primary driver of the species’ disease susceptibility as initially hypothesised. Instead, the interspecific diversity introduced by *E. macquarii* across the genome more broadly, including other variant types not explored in this study, may contribute to the disease resilience of backcross individuals compared to their pure *M. georgesi* counterparts; however, this would need further investigation (Parrish et al. 2024; Yang et al. 2024). Individuals from the after disease group exhibit a reduced number of SNPs, non-synonymous mutations, and alleles at six out of the 15 MHC genes when compared to the before group. These observations may reflect a possible sampling artifact or could represent early evidence of allele loss in the wild, with some of the remaining alleles potentially conferring resistance to nidovirus infection, as seen in amphibians with chytridiomycosis (Fu et al. 2023). However, longer-term monitoring of subsequent generations in addition to metadata of resilient and animals that succumb to the disease will be necessary to validate these findings.

The results of this study should be interpreted with consideration of its limitations. The first limitation is the scarcity of complete coding sequences for testudines and other reptilian species, which hinders the ability to draw high-resolution conclusions about the evolutionary histories and classification of MHC homologs in *M. georgesi* and testudines more broadly. Compared to the extensive genomic and immunological resources available for other tetrapod clades, such as marsupials (Belov et al. 2013), reptiles—particularly testudines—still lack comprehensive immune gene resources. This deficiency in data restricts the ability to resolve evolutionary relationships of ancient lineages effectively. As next-generation sequencing (NGS) becomes more common for non-model organisms, the increasing availability of genomic data and resources will help address the current deficiency in MHC nomenclature and classification. Secondly, are comparisons between pure and backcross animals. Determining whether discrepancies between pure and backcross animals arise from misalignments due to structural variations or interference from paralogous sequences affecting accurate sequence alignment across backcross genomes can be difficult with short-read data. Synteny analyses between pure species could offer valuable insights into chromosomal rearrangements and regions with complex architecture, including the terminal region of scaffold 10 (Li et al. 2022). Additionally, this study uses short-read data to focus on biallelic SNPs as proxies for genetic diversity, similar to the approaches undertaken by Stroupe et al. (2022) and Askelson et al. (2023) investigating hybridisation in North American bison (*Bison bison*) and white-breasted nuthatches (*Sitta carolinensis*), respectively. Exploring structural variants and indels represents another promising approach to gain deeper insights into genomic diversity within and between species. These elements can span multiple alleles, genes, and gene regions simultaneously, potentially exerting a more substantial influence on fitness (Wold et al. 2021). For future functional studies, annotating MHC genes in *E. macquarii* and employing targeted amplicon sequencing would facilitate thorough and precise comparisons of the genomic impacts of hybridisation within MHC loci, including duplications, copy number variations, and expansions in gene families (Horton et al. 2004). Additionally, greater sequencing effort of first-generation hybrids and additional backcross individuals will offer a comprehensive view of genomic dynamics, facilitating a deeper understanding of the extent of introgression and its potential implications for genome-wide and immune function, and overall fitness across a larger sample set.

## Conclusion

Our study demonstrates a high level of conservation in the functionally important MHC core region, in contrast to low, putatively neutral, genome-wide diversity of this critically endangered turtle. Using short-read Illumina re-sequenced genomes, we reconstructed the demographic history of the population, revealing a pronounced and ongoing gradual decline in effective population size and a persistent trajectory towards genetic depletion over time. Genome-wide diversity shows evidence of having diminished over time and is expected to decline further, particularly following the population crash triggered by the 2015 nidovirus outbreak. Lastly, by characterising the MHC region in a freshwater turtle species, this study provides important insights to support ongoing research within this species and lays the groundwork for future investigations into MHC diversity across testudines more broadly.

## Supplementary Information

Below is the link to the electronic supplementary material.

Supplementary file1 (PDF 1156 KB)

## Data Availability

The male *M. georgesi* reference genome assembly, all raw sequencing reads including the 3-tissue transcriptome RNA-seq reads, whole-genome re-sequencing data and annotated sequences are available from NCBI under BioProject PRJNA1003540. All scripts used to process data are publicly available at https://github.com/awgg-lab/australasiangenomes
