# Supplementary material for: Genome-wide diversity and MHC characterisation in a critically endangered freshwater turtle susceptible to disease
Source: Immunogenetics. 2025 May 6;77(1):21. doi: 10.1007/s00251-025-01378-8 (PMC12055648; doi:10.1007/s00251-025-01378-8)
Supplement: Supplementary file 1 — Supplementary file1 (PDF 1156 KB) [file 251_2025_1378_MOESM1_ESM.pdf]

Genome-wide diversity and MHC characterisation in a critically endangered freshwater turtle susceptible to disease

Supplementary Material

Title: Genome-wide diversity and MHC characterisation in a critically endangered freshwater turtle susceptible to disease
Journal: *Immunogenetics*
Authors: Holly V. Nelson[1], Luke W. Silver[1,2], Toby G. L. Kovacs[1], Elspeth A. McLennan[1], Arthur Georges[3], Jane L. DeGabriel[4], Carolyn J. Hogg[1,2*] and Katherine Belov[1,2]
Author affiliations: [1] School of Life and Environmental Sciences, The University of Sydney, Sydney, NSW 2006, Australia.
[2] Australian Research Council Centre of Excellence for Innovations in Peptide and Protein
[3] Institute for Applied Ecology, University of Canberra, Bruce, ACT 2617, Australia
Science, The University of Sydney, Sydney, NSW 2006, Australia
[4] NSW Department of Climate Change, the Environment, Energy and Water, Parramatta, NSW 2150, Australia
Corresponding author: Professor Carolyn Hogg | carolyn.hogg@sydney.edu.au

Table S1. Reference species and sequences for Major Histocompatibility Complex (MHC) BLAST search in *Myuchelys georgesi*. Complete coding sequences (complete) were used when available. Partial coding sequences (partial) were also included. The corresponding MHC class and shared common ancestor group are also listed.

**Class**	**Reference MHC species**	**Project**	**Sequence type**	**Shared taxonomic group**
**MHC I**	Tawny dragon (*Ctenophorus decresii*)	KY905241.1	Complete	Class Reptilia
**MHC I**	Caiman (*Caiman crocodilus*)	KF769542.1	Complete	Class Reptilia
**MHC I**	Marine iguana (*Amblyrhynchus cristatus*)	EU839663.1	Complete	Class Reptilia
**MHC I**	Galapagos land iguana (*Conolophus subcristatus*)	EU604313.1	Complete	Class Reptilia
**MHC I**	Tuatara (*Sphenodon punctatus*)	DQ145788.1	Complete	Class Reptilia
**MHC I**	Green sea turtle (*Chelonia mydas*)	OK135213.1	Partial	Order testudines
**MHC II**	Chinese softshell turtle (*Pelodiscus sinensis*)	MT834970.1	Complete	Order testudines
**MHC II**	Marine iguana (*Amblyrhynchus cristatus*)	FJ623752.1	Complete	Order Reptilia
**MHC II**	Green-rumped parrotlet (*Forpus passerines*)	EF710746.1	Partial	Sauropsida

Table S2. Genbank Accession numbers of complete MHC I sequences used for phylogenetic comparisons.

**Accession**	**Species**	**Common name**	**Classification**	**Name on tree**
L20733.1	*Xenopus laevis*	African clawed frog	Amphibia	*Xela-U*
HQ158307.1	*Crocodylus porosus*	Saltwater crocodile	Crocodilia	*Crpo-U*
KF769542.1	*Caiman crocodilus*	Caiman	Crocodilia	*Cacr-U*
JU175072.1	*Crotalus adamanteus*	Eastern Diamondback rattlesnake	Squamata	*Crad-U*
KM515952.1	*Tiliqua rugosa*	Shingleback	Squamata	*Tiru-UB*
KY905241.1	*Ctenosaura decressii*	Tawny dragon	Squamata	*Ctde-UA*
EU604313.1	*Conolophus subcristatus*	Galapagos iguana	Squamata	*Cosu-UB*
EU604308.1	*Amblyrhynchus cristatus*	Marine iguana	Squamata	*Amcr-UB*
DQ145788.1	*Sphenodon punctatus*	Tuatara	Rhynchocephalia	*Sppu-U*01*
DQ145789.1	*Sphenodon punctatus*	Tuatara	Rhynchocephalia	*Sppu-U*02*
X12780.1	*Gallus gallus*	Chicken	Aves	*Gaga_F10*
MT260395.1	*Anas platyrhynchos*	Mallard	Aves	*Anpl-UAA*
XM_043527918.1	*Chelonia mydas*	Green sea turtle	Testudines	*Chmy-U*
XM_048817647.1 425-703	*Caretta caretta*	Loggerhead sea turtle	Testudines (partial predicted)	*Caca-F10.1*
XM_048817647.1 163-424	*Caretta caretta*	Loggerhead sea turtle	Testudines (partial predicted)	*Caca-F10.2*
XM_048817647.1 163-424	*Terrapene carolina triunguis*	Three-toed box turtle	Testudines (partial predicted)	*Teca-F10.1*
XM_029914387.1 77-337	*Terrapene carolina triunguis*	Three-toed box turtle	Testudines (partial predicted)	*Teca-F10.2*
MN339476.1	*Ginglymostoma cirratum*	Nurse shark	Orectolobiformes	*Gici-UDA*

Table S3. Genbank Accession numbers of complete and partial (indicated by *) MHC II beta sequences used for phylogenetic comparisons.

**Accession**	**Species**	**Common name**	**Classification**	**Name on tree**
KP118841.1	*Crocodylus porosus*	Saltwater crocodile	Crocodilia	*Crpo-DAB1*
KP118846.1	*Crocodylus porosus*	Saltwater crocodile	Crocodilia	*Crpo-DAB2*
AF256652.1	*Caiman crocodilus*	Caiman	Crocodilia	*Cacr-beta*
XM_008121064.1	*Anolis carolinensis*	Green anole	Squamata	*Anca-beta*
FJ623746.1	*Amblyrhynchus cristatus*	Marine iguana	Squamata	*Amcr-DAB1*
FJ623750.1	*Amblyrhynchus cristatus*	Marine iguana	Squamata	*Amcr-DAB3*
DQ124231.1	*Sphenodon punctatus*	Tuatara	Rhynchocephalia	*Sppu-DAB1*
DQ124232.1_	*Sphenodon punctatus*	Tuatara	Rhynchocephalia	*Sppu-DAB2*
DQ124233.1	*Sphenodon punctatus*	Tuatara	Rhynchocephalia	*Sppu-DBB*
NM_001312902.2	*Gallus gallus*	Chicken	Aves	*Gaga-DMB1*
KJ162461.1	*Columba livia*	Rock dove	Aves	*Coli-DAB*
EU442606.2	*Tyto alba*	Barn owl	Aves	*Tyal-DAB1*
PQ368796.1*	*Dermochelys coriacea*	Leatherback sea turtle	Testudines	*Deco-B01*
PQ228150.1*	*Dermochelys coriacea*	Leatherback sea turtle	Testudines	*Deco-B20*
PQ368795.1*	*Chelonia mydas*	Green sea turtle	Testudines	*Chmy-B*
AY937206.1*	*Mauremys reevesii*	Chinese pond turtle	Testudines	*Mare-B*
PQ368797.1*	*Lepidochelys kempii*	Kemp’s ridley sea turtle	Testudines	*Leke-B*
PQ368784.1*	*Caretta caretta*	Loggerhead sea turtle	Testudines	*Caca-B*
KU949601.1*	*Gopherus polyphemus*	Gopher tortoise	Testudines	*Gopo-B*
MN364682.1	*Alosa sapidissima*	American Shad	Clupeiformes	*Alsa-beta*

Table S4. Metadata of samples used for analyses. MG *Myuchelys georgesi,* EM *Emydura macquarii,* WGR Whole genome resequencing, DIN DNA Integrity Number, WG Whole genome, MHC Major histocompatibility complex.

**Specimen ID**	**Sample ID**	**Sample type**	**Species**	**Year**	**Group**	**Data type**	**DIN**	**Total number of reads**	**Alignment rate (%)**	**X Coverage (WG)**	**X Coverage (MHC core region)**
UC<Aus>AA036038	AA036038	skin	MG	2007	Before	WGR	8.6	327,259,209	99.17	24.0832	22.26
UC<Aus>AA036098	AA036098	skin	MG	2007	Before	WGR	8.8	378,250,227	98.67	26.9741	32.0915
UC<Aus>AA036219	AA036219	skin	MG	2007	Before	WGR	8.4	405,089,073	99.25	29.5810	29.3836
UC<Aus>AA036245	AA036245	skin	MG	2007	Before	WGR	9.2	378,250,227	98.65	27.8552	32.0915
UC<Aus>AA036250	AA036250	skin	MG	2007	Before	WGR	7.7	271,276,297	99.37	20.0679	19.4859
UC<Aus>AA036253	AA036253	skin	MG	2007	Before	WGR	8.1	308,343,699	99.01	23.4763	24.7508
UC<Aus>AA036275	AA036275	skin	MG	2007	Before	WGR	8.0	402,251,577	99.59	29.5515	27.131
UC<Aus>AA036814	AA036814	skin	MG	2007	Before	WGR	8.8	340,200,198	99.55	24.8715	30.1402
UC<Aus>AA036815	AA036815	skin	MG	2007	Before	WGR	7.9	393,793,342	99.51	29.5132	36.0545
UC<Aus>AA036831	AA036831	skin	MG	2007	Before	WGR	8.9	382,913,188	99.50	28.3368	30.3626
UC<Aus>AA036869	AA036869	skin	MG	2007	Before	WGR	8.5	424,026,092	99.46	31.1349	34.2918
UC<Aus>AA036889	AA036889	skin	MG	2007	Before	WGR	9.0	336,208,149	99.48	24.4811	27.8062
UC<Aus>AA048006	AA048006	skin	MG	2007	Before	WGR	8.8	311,804,759	98.97	22.5681	21.2803
UC<Aus>AA048016	AA048016	skin	MG	2007	Before	WGR	9.0	291,248,743	98.38	21.1652	20.7545
UC<Aus>AA048033	AA048033	skin	MG	2007	Before	WGR	8.3	321,311,371	99.23	23.4815	19.4373
UC<Aus>AA048041	AA048041	skin	MG	2007	Before	WGR	8.1	338,114,713	99.56	24.7781	21.483
UC<Aus>AA048057	AA048057	skin	MG	2007	Before	WGR	9.2	360,849,823	99.54	26.5659	26.0026
UC<Aus>AA048084	AA048084	skin	MG	2007	Before	WGR	7.5	416,693,738	99.53	30.4186	23.7254
UC<Aus>AA048177	AA048177	skin	MG	2007	Before	WGR	8.0	225,531,249	99.40	26.0214	16.4083
UC<Aus>BRST_5085	BRST_5085_04	Blood stain	MG	2019	After	WGR	6.8	351,905,938	99.63	25.3754	30.2449
UC<Aus>BRST_5246	BRST_5246_01	Blood stain	MG	2019	After	WGR	6.5	361,674,761	99.67	26.5123	24.4038

UC<Aus>BRST_5247	BRST_5247_01	Blood stain	MG	2019	After	WGR	6.9	363,328,046	99.66	26.2223	22.1225
UC<Aus>BRST_5248	BRST_5248_01	Blood stain	MG	2019	After	WGR	6.8	320,639,849	99.65	23.4789	21.1071
UC<Aus>BRST_5252	BRST_5252_01	Blood stain	MG	2019	After	WGR	7.1	383,226,426	99.63	28.2814	23.9447
UC<Aus>BRST_6013	BRST_6013_01	Blood stain	MG	2019	After	WGR	6.9	375,063,401	99.65	27.6898	25.0523
UC<Aus>BRST_6017	BRST_6017_01	Blood stain	MG	2019	After	WGR	6.8	368,651,967	99.54	27.0532	22.8837
UC<Aus>BRST_6023	BRST_6023_01	Blood stain	MG	2019	After	WGR	7.1	317,510,219	99.62	23.2046	21.9703
UC<Aus>BRST_6024	BRST_6024_01	Blood stain	MG	2019	After	WGR	7.3	300,456,627	99.63	21.7630	16.1012
UC<Aus>BRST_6093	BRST_6093_01	Blood stain	MG	2019	After	WGR	6.8	381,823,641	99.63	27.5621	30.5983
UC<Aus>BRST_6095	BRST_6095_01	Blood stain	MG	2019	After	WGR	6.1	354,938,847	99.59	25.8669	28.7898
UC<Aus>BRST_6115	BRST_6115_01	Blood stain	MG	2019	After	WGR	6.3	322,128,401	99.01	25.3149	22.921
UC<Aus>BRST_6096	BRST_6096_01	Blood stain	MG x EM	2019	Backcross	WGR	6.3	383,362,349	99.57	29.7850	44.7904
UC<Aus>BRST_5251	BRST_5251_02	Blood stain	MG x EM	2019	Backcross	WGR	6.7	400,084,195	99.55	31.4142	28.1824
UC<Aus>BRST_6029	BRST_6029_01	Blood stain	MG x EM	2019	Backcross	WGR	6.2	425,733,383	99.52	32.4808	41.7436
UC<Aus>BRST_6099	BRST_6099_01	Blood stain	MG x EM	2019	Backcross	WGR	6.2	481,971,383	99.52	37.4561	50.9024

Table S5: Complete list of annotated MHC exons in genomic order along chromosome 10, based on whole genome coordinates from the reference genome (GCA_040894355.1). ORF: Open Reading Frame.

**Class**	**Gene**	**ORF length (bp)**	**Exon total**	**Exon**	**Start**	**End**	**Strand**
**MHC I**	*Myge-UB*	1124	8	8	30471574	30471666	-
				7	30473115	30473143	-
				6	30473497	30473530	-
				5	30474174	30474264	-
				4	30474404	30474680	-
				3	30475552	30475826	-
				2	30488887	30489157	-
				1	30493423	30493484	-
	Myge-UE	1196	7	7	30500310	30500506	-
				6	30500690	30500713	-
				5	30501301	30501391	-
				4	30501531	30501807	-
				3	30502340	30502614	-
				2	30503854	30504130	-
				1	30508464	30508525	-
	Myge-UC	1096	7	7	30533405	30533478	-
				6	30533917	30533949	-
				5	30534318	30534434	-
				4	30534837	30535115	-
				3	30535937	30536212	-
				2	30537832	30538093	-
				1	30538879	30538940	-
	Myge-UD	1063	7	7	30567953	30567993	-
				6	30568557	30568589	-
				5	30568935	30569051	-
				4	30569521	30569799	-
				3	30570806	30571081	-
				2	30572447	30572708	-
				1	30577604	30577665	-
	Myge-UA	1063	7	7	30583497	30583537	-
				6	30584108	30584140	-
				5	30584486	30584602	-
				4	30585060	30585337	-
				3	30587357	30587633	-
				2	30588820	30589081	-
				1	30591307	30591368	-
**MHC II**	*Myge-DAA1*	774	4	4	30660982	30661136	-
				3	30661272	30661554	-
				2	30662018	30662275	-
				1	30663406	30663487	-
	Myge-DAB1	805	6	1	30665199	30665305	+
				2	30665695	30665968	+
				3	30666785	30667061	+
				4	30668402	30668513	+

			5	30669052	30669077	+
			6	30669438	30669452	+
Myge-DAA2	774	4	4	30677698	30677852	-
			3	30677989	30678271	-
			2	30678664	30678921	-
			1	30680142	30680223	-
Myge-DAB2	807	6	1	30681654	30681760	+
			2	30682482	30682752	+
			3	30683567	30683849	+
			4	30685180	30685291	+
			5	30685795	30685819	+
			6	30686179	30686193	+
Myge-DAA3	774	4	4	30698489	30698644	-
			3	30698780	30699061	-
			2	30699528	30699785	-
			1	30700907	30700988	-
Myge-DAB3	804	6	1	30702397	30702503	+
			2	30702901	30703174	+
			3	30704096	30704372	+
			4	30705339	30705450	+
			5	30706005	30706029	+
			6	30706334	30706348	+
Myge-DAA4	775	4	4	30717695	30717850	-
			3	30717983	30718265	-
			2	30718651	30718908	-
			1	30720110	30720191	-
Myge-DAB4	807	6	1	30721446	30721552	+
			2	30721861	30722131	+
			3	30722953	30723235	+
			4	30724562	30724673	+
			5	30725144	30725168	+
			6	30725528	30725542	+
Myge-DAA5	751	4	4	30735202	30735357	-
			3	30735492	30735774	-
			2	30736241	30736498	-
			1	30737725	30737782	-
Myge-DAB5	777	5	1	30739700	30739806	+
			2	30740419	30740689	+
			3	30741306	30741582	+
			4	30742814	30742925	+
			5	30743771	30743785	+

Table S6: Pairwise differentiation statistics between exonic nucleotide sequences of (A) MHC I and (B) MHC II *Myge* genes, generated using EMBL-EBI Clustal Omega.

(A)

	UA	*UB*	*UC*	*UD*	*UE*
UA	0				
UB	0.523	0			
UC	0.115	0.474	0		
UD	0.134	0.541	0.120	0	
UE	0.506	0.187	0.495	0.541	0

(B)

	DAA1	*DAB1*	*DAA2*	*DAB2*	*DAA3*	*DAB3*	*DAA4*	*DAB4*	*DAA5*	*DAB5*
DAA1	0									
DAB1	0.421	0								
DAA2	0.088	0.448	0							
DAB2	0.503	0.093	0.48	0						
DAA3	0.046	0.481	0.081	0.510	0					
DAB3	0.494	0.083	0.499	0.069	0.493	0				
DAA4	0.090	0.571	0.092	0.505	0.076	0.591	0			
DAB4	0.474	0.118	0.478	0.103	0.497	0.112	0.504	0		
DAA5	0.060	0.450	0.077	0.507	0.064	0.493	0.094	0.502	0	
DAB5	0.467	0.104	0.492	0.080	0.559	0.101	0.521	0.095	0.485	0

Table S7: Summary of runs of homozygosity (ROH) metrics for individuals in Before and After groups. Each row represents a different individual with details on total ROH length; percentage of genomic segments in ROH; FROH (proportion of the genome in ROH); Short FROH (<2mb); and long FROH (>2mb). π genome-wide nucleotide diversity including ROH; and number of genome-wide SNPs generated using BCFtools 'stats' on BAM files.

Individual	ID	Group	Segments in ROH (%)	Total ROH Length (bp)	FROH	FROH < 2Mb	FROH > 2Mb	π	Genome-wide SNPs
**1**	AA036038	Before	91.44	1,245,800,000	0.881166358	0.112745158	0.768421201	8.00068e-05	635,644
**2**	AA036098	Before	90.54	1,235,700,000	0.874022531	0.129296203	0.744726327	8.00148e-05	678,942
**3**	AA036219	Before	90.39	1,232,700,000	0.871900602	0.100579432	0.77132117	8.00064e-05	644,948
**4**	AA036245	Before	90.56	1,235,600,000	0.8739518	0.129084011	0.744867789	8.00042e-05	678,942
**5**	AA036250	Before	92.43	1,266,200,000	0.895595475	0.077167482	0.818427993	8.00008e-05	596,722
**6**	AA036253	Before	91.58	1,247,900,000	0.882651709	0.096264843	0.786386866	8.00165e-05	603,276
**7**	AA036275	Before	90.16	1,231,200,000	0.870839637	0.104540366	0.766299272	8.00015e-05	689,602
**8**	AA036814	Before	91.89	1,257,700,000	0.889583343	0.10192332	0.787660023	8.00000e-05	628,311
**9**	AA036815	Before	90.66	1,228,800,000	0.869142094	0.120525564	0.74861653	8.00026e-05	628,311
**10**	AA036831	Before	89.76	1,216,500,000	0.860442186	0.12901328	0.731428906	8.00014e-05	670,108
**11**	AA036869	Before	92.13	1,258,700,000	0.890290653	0.099023351	0.791267302	8.0002e-05	685,019
**12**	AA036889	Before	90.13	1,227,700,000	0.868364054	0.112391503	0.755972551	8.00006e-05	675,974
**13**	AA036889	Before	90.98	1,240,400,000	0.877346886	0.125971848	0.751375038	8.00028e-05	645,123
**14**	AA048016	Before	90.87	1,245,700,000	0.881095627	0.115645127	0.7654505	8.00083e-05	644,116
**15**	AA048033	Before	91.58	1,255,800,000	0.888239455	0.094496569	0.793742886	8.00001e-05	637,253
**16**	AA048041	Before	91.13	1,245,700,000	0.881095627	0.105389137	0.77570649	8.00001e-05	652,759

**17**	AA048057	Before	91.61	1,249,900,000	0.884066328	0.105106214	0.778960114	8.00029e-05	661,346
**18**	AA048084	Before	90.89	1,238,100,000	0.875720074	0.10927934	0.766440734	8.00019e-05	660,667
**19**	AA048177	Before	91.11	1,245,800,000	0.881166358	0.104469635	0.776696723	8.00005e-05	577,242
**20**	MG6143	After	90.34	1,227,600,000	0.868293323	0.119676792	0.74861653	8.00076e-05	759,331
**21**	MG5085	After	91.55	1,248,500,000	0.883076094	0.105035483	0.778040612	8.00014e-05	678,949
**22**	MG5246	After	90.42	1,235,400,000	0.873810338	0.107723259	0.766087079	8.00017e-05	699,762
**23**	MG5247	After	92.02	1,258,900,000	0.890432115	0.103479401	0.786952713	8.00063e-05	681,937
**24**	MG5248	After	91.01	1,238,300,000	0.875861536	0.0945673	0.781294236	8.00029e-05	668,178
**25**	MG5252	After	90.58	1,239,000,000	0.876356653	0.099589198	0.776767454	8.00075e-05	754,239
**26**	MG6013	After	90.69	1,233,600,000	0.872537181	0.128023046	0.744514134	8.00034e-05	690,871
**27**	MG6017	After	90.33	1,232,900,000	0.872042064	0.108359838	0.763682226	8.00026e-05	674,961
**28**	MG6023	After	90.39	1,230,700,000	0.870485983	0.125476731	0.745009251	8.00114e-05	705,073
**29**	MG6024	After	91.35	1,254,900,000	0.887602876	0.10121601	0.786386866	8.00009e-05	657,418
**30**	MG6093	After	91.24	1,244,700,000	0.880388318	0.106450102	0.773938216	8.00017e-05	723,806
**31**	MG6095	After	91.21	1,247,900,000	0.882651709	0.10920861	0.773443099	8.00002e-05	719,412
**32**	MG5251	F2 Backcross	0.057	700,000	0.000495117	0.000495117	0	0.00484852	15,307,413
**33**	MG6029	F2 Backcross	0.021	200,000	0.000141462	0.000141462	0	0.00485748	11,562,932
**34**	MG6096	F2 Backcross	0.071	800,000	0.000565848	0.000565848	0	0.00442102	13,309,234
**35**	MG6099	F2 Backcross	0.035	400,000	0.000282924	0.000282924	0	0.00483882	15,218,993

Table S8. Summary of SNP dropout when filtering at different minor allele frequency (MAF) thresholds.

**MAF filter**	**Variants**	**Percent**
<0.05	3354	0.09%
<0.04	2979	0.08%
<0.03	2559	0.07%
<0.02	2545	0.07%
<0.01	1903	0.05%
Total	3,681,055	100%

Table S9: MHC I and II heterozygosity statistics for Before (N = 19) and After (N=12) groups. Observed (H_O) and expected (H_E) heterozygosity calculated using GenAlEx v6.5. "All" refers to statistics calculated across all exons for the respective class. SE indicates standard error.

		**Before**		**After**	
		H_O (± SE)	H_E (± SE)	H_O (± SE)	H_E (± SE)
**MHC I**	*Myge-UA*	0.177 (0.037)	0.211 (0.003)	0.169 (0.051)	0.210 (0.000)
	Myge-UB	0.014 (0.004)	0.058 (0.000)	0.008 (0.005)	0.058 (0.000)
	Myge-UC	0.047 (0.015)	0.024 (0.012)	0.024 (0.012)	0.018 (0.000)
	Myge-UD	0.039 (0.009)	0.109 (0.000)	0.037 (0.011)	0.039 (0.008)
	Myge-UE	0.218 (0.049)	0.249 (0.000)	0.211 (0.062)	0.249 (0.000)
	All	0.118 (0.018)	0.148 (0.001)	0.100 (0.022)	0.147 (0.001)
**MHC II**	*Myge-DAA1*	0.014 (0.003)	0.056 (0.000)	0.029 (0.015)	0.056 (0.000)
	Myge-DAB1	0.014 (0.002)	0.056 (0.000)	0.029 (0.015)	0.056 (0.000)
	Myge-DAA2	0.007 (0.002)	0.050 (0.000)	0.006 (0.001)	0.050 (0.000)
	Myge-DAB2	0.214 (0.043)	0.229 (0.000)	0.231 (0.054)	0.229 (0.000)
	Myge-DAA3	0.009 (0.002)	0.076 (0.002)	0.007 (0.002)	0.075 (0.002)
	Myge-DAB3	0	0.069 (0.000)	0	0.068 (0.000)
	Myge-DAA4	0.135 (0.039)	0.192 (0.002)	0.123 (0.039)	0.194 (0.002)
	Myge-DAB4	0.132 (0.031)	0.184 (0.001)	0.120 (0.035)	0.181 (0.003)
	Myge-DAA5	0.227 (0.033)	0.196 (0.000)	0.176 (0.042)	0.196 (0.000)
	Myge-DAB5	0.137 (0.0307)	0.163 (0.000)	0.127 (0.038)	0.163 (0.000)
	All	0.122 (0.019)	0.148 (0.001)	0.102 (0.023)	0.147 (0.001)

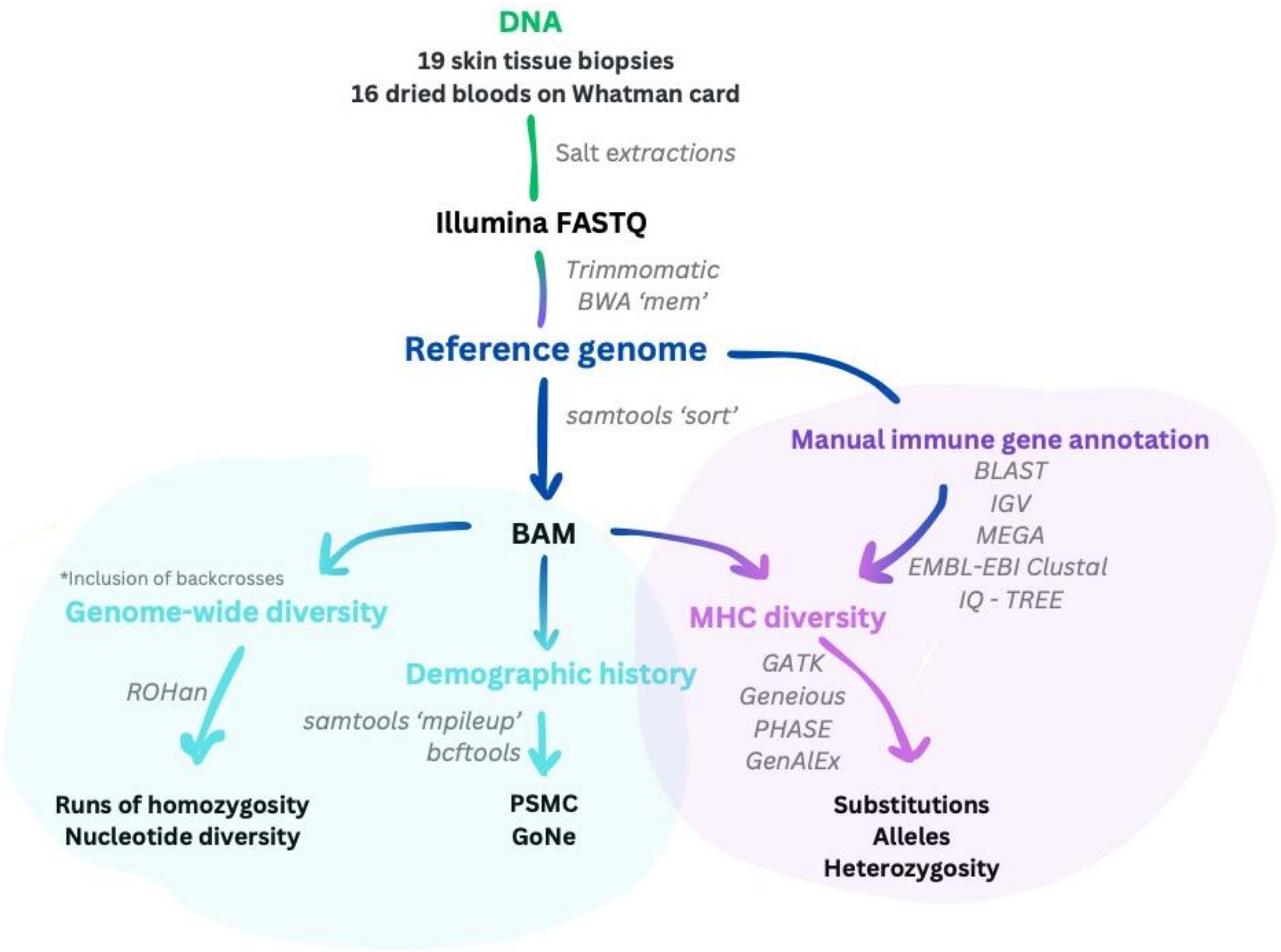

Figure S1. A flowchart outlining the methodological approaches used in this study. Blue highlights genome-wide neutral diversity investigations and purple highlights MHC gene annotation characterisation.

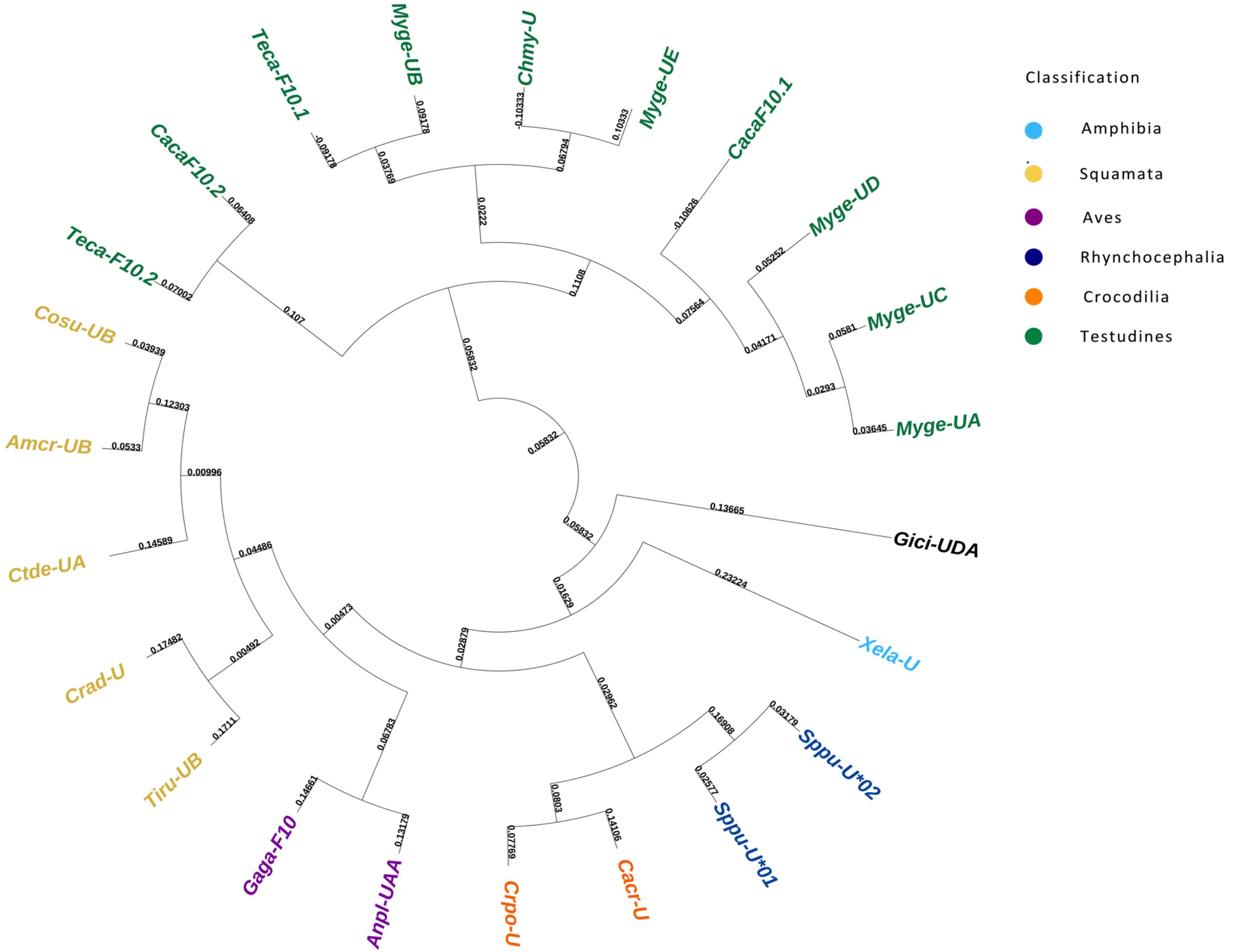

Figure S2. Maximum likelihood analysis of MHC I sequences for non-avian and avian reptiles. Branch lengths based on 1000 replicates are shown. Nurse shark (*Ginglymostoma cirratum*) (*Gici-UDA*) was used as an outgroup.

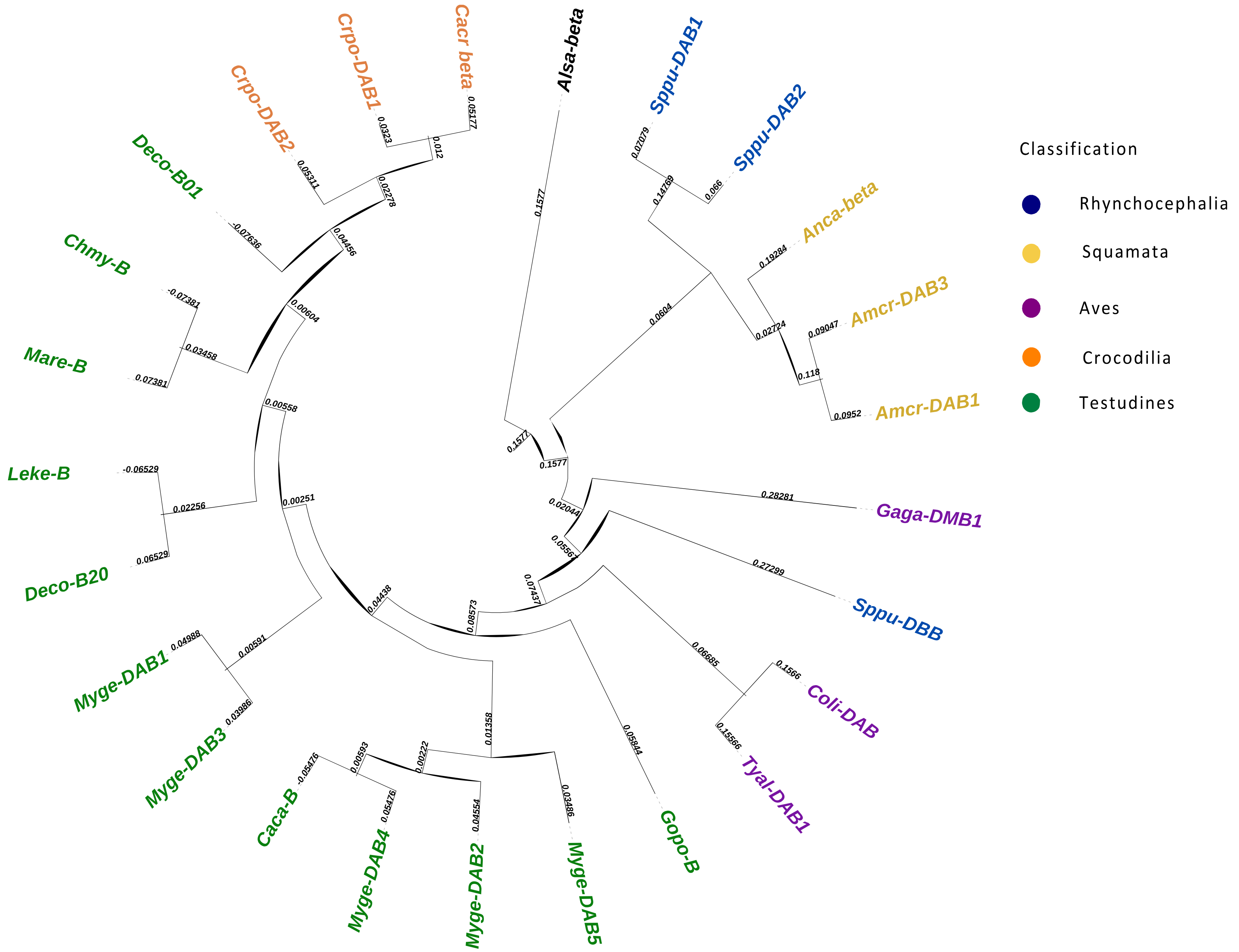

Figure S3: Maximum likelihood analysis of complete MHC II beta sequences for non-avian and avian reptiles. Branch lengths based on 1000 replicates are shown. American shad (*Alosa sapidissima*) (*Alsa-beta*) was used as an outgroup. Chicken DMB and Tuatara DBB sequences were included for DMB and DBB-like clades.

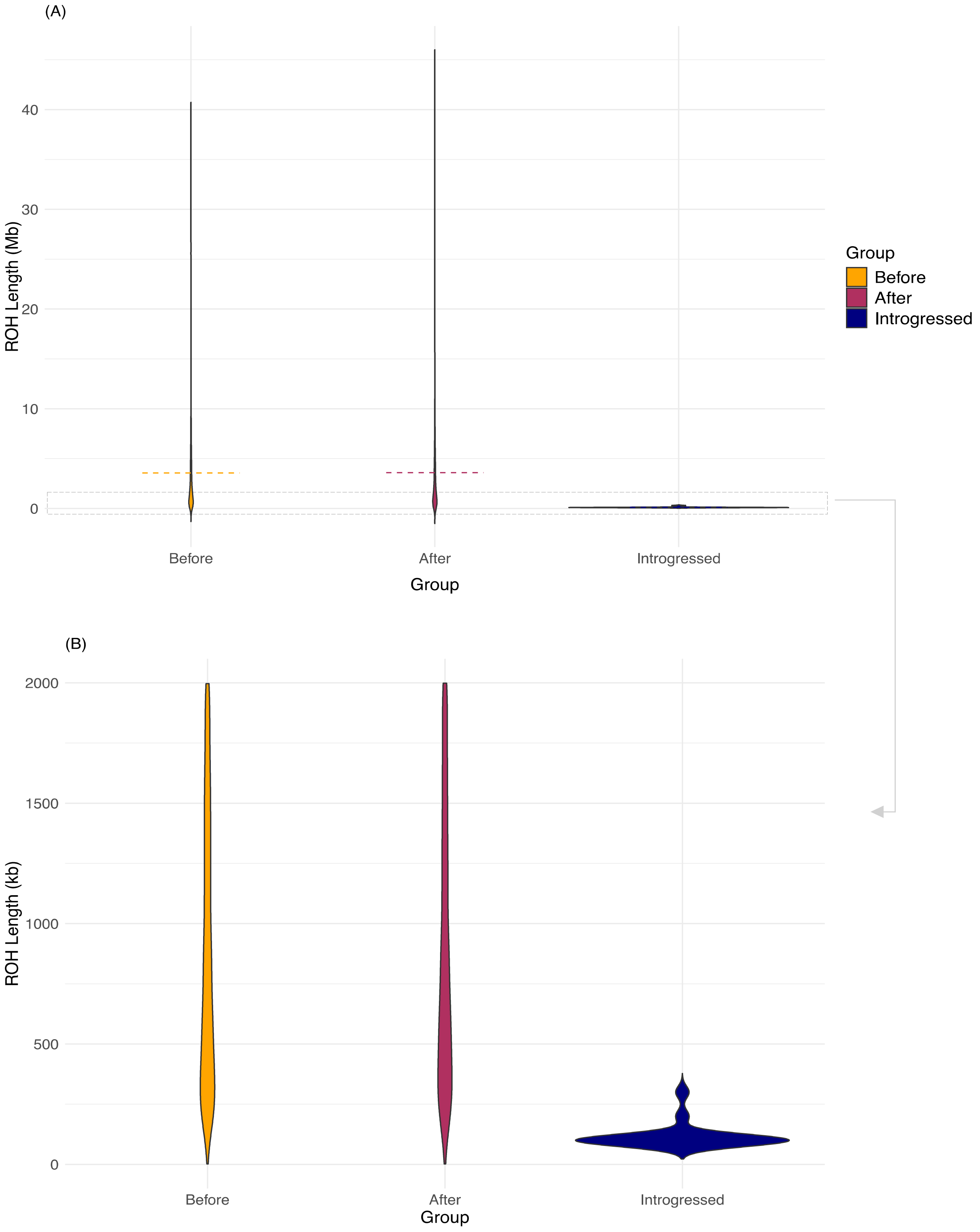

Figure S4: Violin plots showing the distribution of run lengths of homozygosity (ROHs) calculated using ROHan across the Before, After, and Backcross groups. (A) Distribution of all ROH lengths, with a dotted line indicating the mean ROH length. (B) An enlarged view showing distribution of short ROHs with lengths less than 2 Mb.

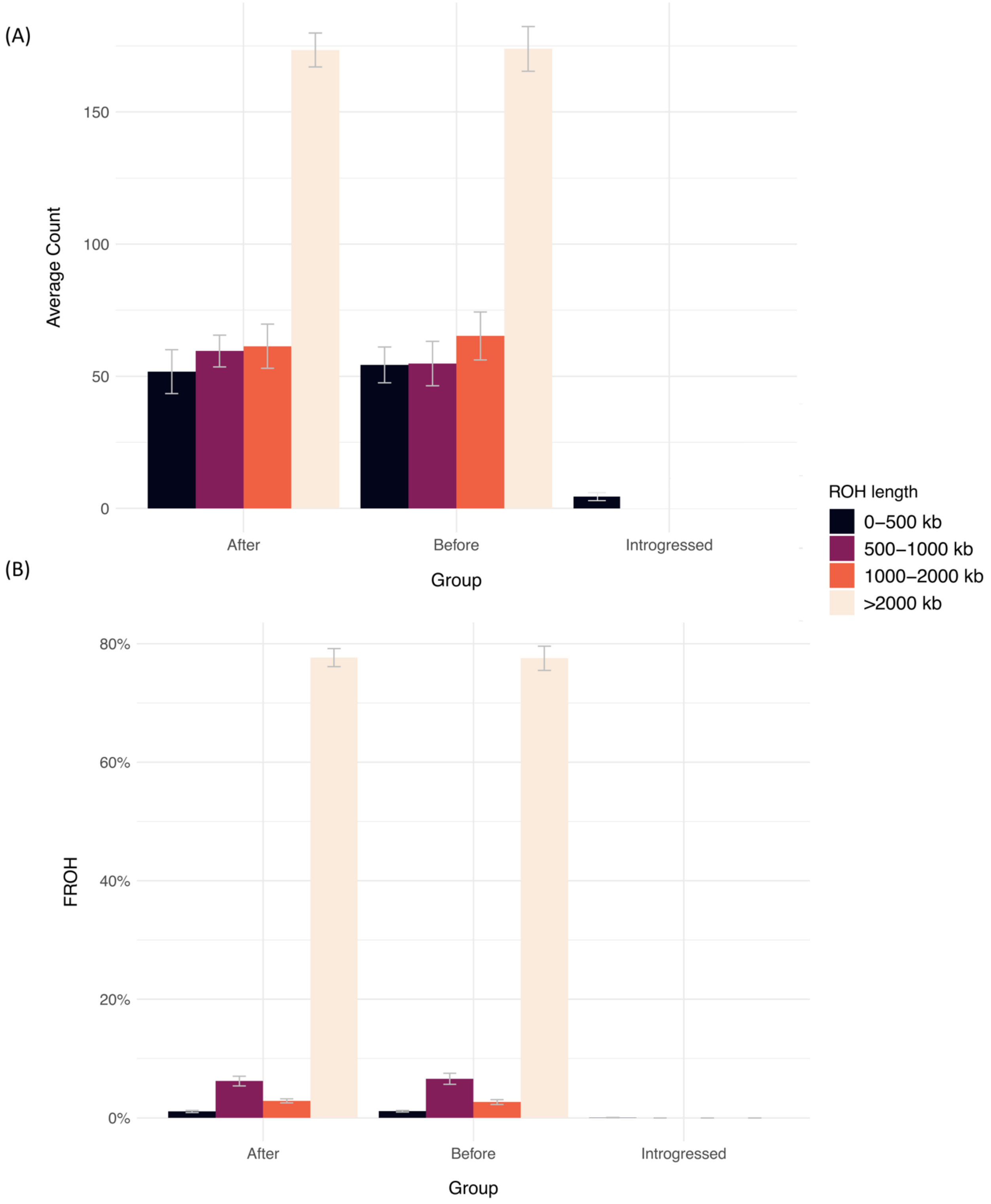

Figure S5. (A) Average count of runs of homozygosity (ROH) across 0-500 kb, 500-1000 kb, 1000-2000 kb, and >2000 kb length categories for individuals in Before, After, and backcross groups. (B) Inbreeding coefficient based on ROH (F_{ROH}) across 0-500 kb, 500-1000 kb, 1000-2000 kb, and >2000 kb length categories for individuals in Before, After, and backcross groups. The F_{ROH} is represented as a percentage of the total length of the 9 macrochromosomes.

(A)

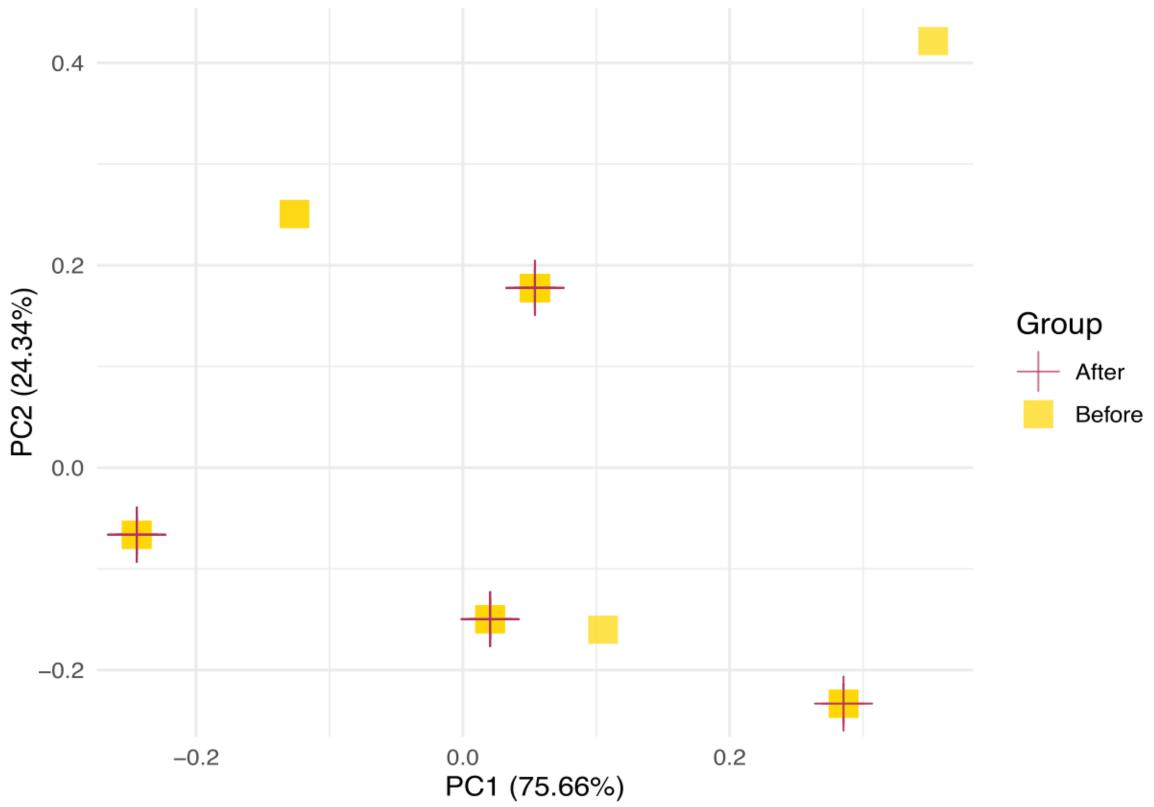

(B)

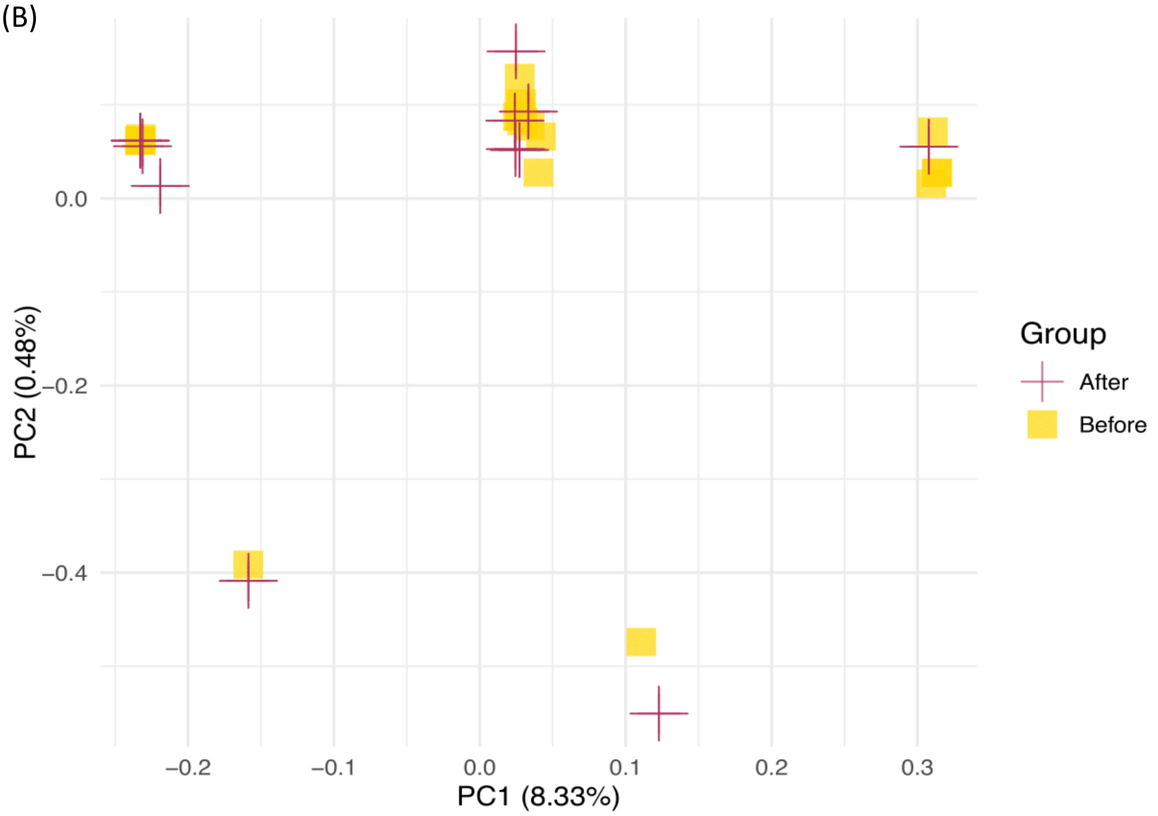

Figure S6. Principal component analysis plots of SNPs in pure *Myuchelys georgesi* from the Before (N = 19) and After (N = 12) groups in (A) MHC I and (B) MHC II.

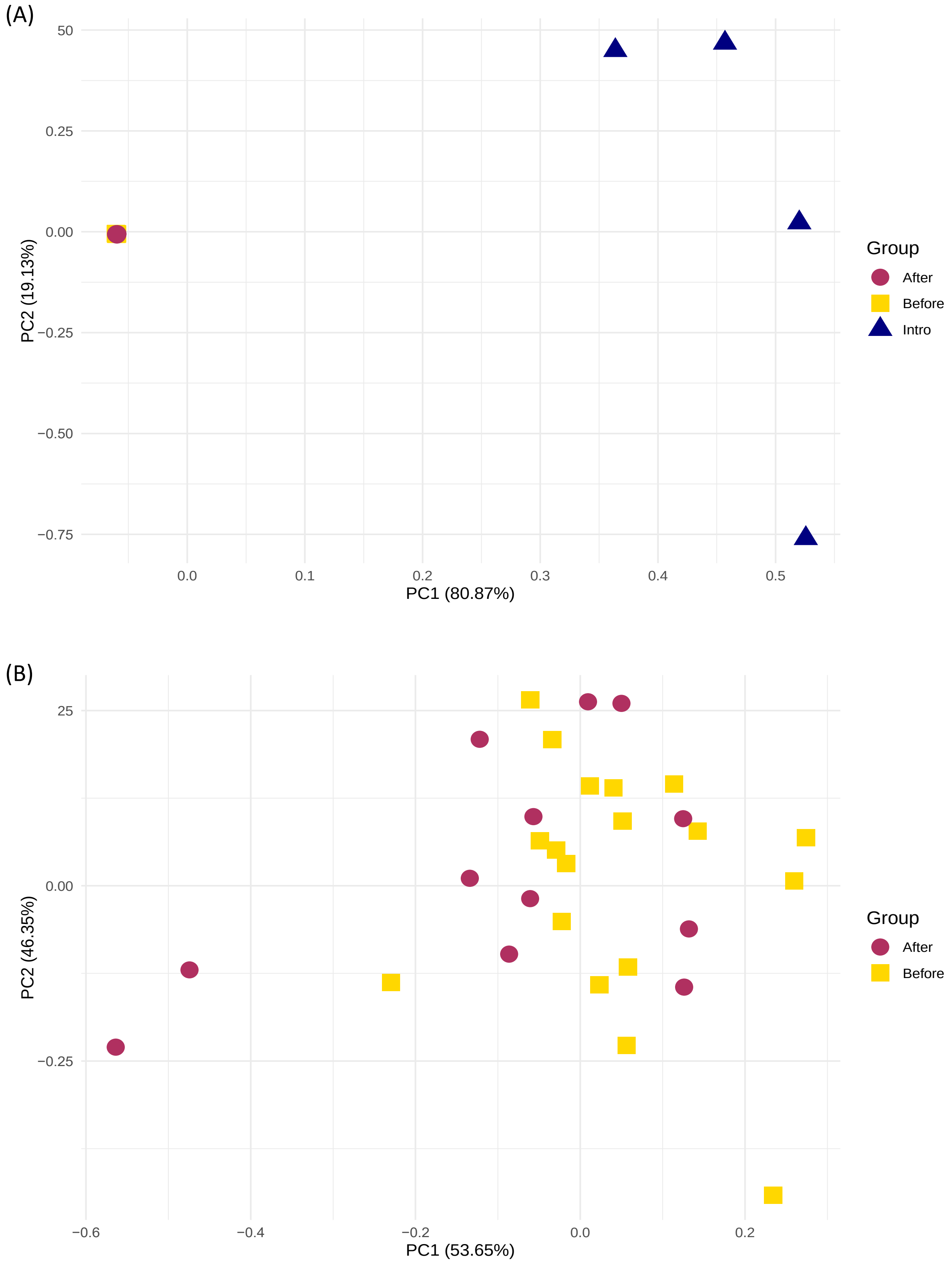

Figure S7. Principal component analysis plots of genome-wide SNPs in (A) pure *Myuchelys georgesi* Before (N = 19), After (N = 12), and F2 backcross animals (N = 4), and (B) pure *M. georgesi* Before (N = 19) and After (N = 12).

All exon sequences for MHC class I genes annotated using the reference genome

> *Myge-UA*
CAGGATGAAGAGCTGGAGGCAGAATCTTCTCCGGTCTTTGGGCTGCCGGAGTGTAGCCGCGTTTGGGGGTGC
TGCTGATTTTCTTCTCCACAGAATGACGCTGGCCCCTACGGCTCCAGCCAGAACCAGCACGGCCAGGACGATG
GCGGCCAGGACCCCAGGGGACAGGGGGCTCTTCTTCCCAGGGGCCAGGCGAGTGTGGGGTCTGATAGGCTG
CTGTGCTCCACCCGGCAGGCATAGCGGTGACTGTCCTCCTGCTGCGGGGAGATCTCCAGGGATGACTGCGTG
TAGTAGGTGCCATCAGCATTGGGCAGGATCCCACTGGAGTGCTTCTCCACCAGGATGTCTTCTCCATCCCGCA
CCCAGGAGACGTGGATGGGACGTGGGTAAAAGCCCCTGGCGCGGCAGGAGAGGGTGACAGAGCTGTCTGT
AGAGTCTGTGTAGGAAACCGAGACCTCGGGGGGTCCTGCTGCTCCAGCACCGTCCTCCCCTGCTGCACCAGG
CTCTGCAGAGTCCCCAGGCACTCATGCTGCAGGTACTGCCGGACAAACTGAGTCCAGGCCTTGCCCGTCTCCC
AGCTCTGCTTCTGGGGGAAGGCCGGCTGCACGGCCGCGACCCACGTCCCCGTCTGGTTGTCAAAGCTGATGA
AGTCCTTCCCGTCGTAGGCATACTGGAACTTCGGGTCCACAAGCACCTGGCCGCTCAGGGCACAGCTCACGTG
AACCTGCTCGGTGTGAATCCCGCCCGTCTGGTTGTGCAGCTGCATCCACCACCTGGTATTGACTTTACAGCCCT
CCTCGTATCCCCAGAACTCCTGGGTCTTTTCCTTGAGATACTCAACACCCACGGCCTGTGTCGCCCAATCCTGG
GTAGGTCTGACCTCTCGCGTGTCGCTGCTGTAGTACGCGATCTTCACGTCATCCAGCTTGGCAATCATGAAGTA
GCGATTGGTCCCCTCCTCGTCCATGACTGCTGTGACTAGCACGGCCAGGCTGTGGTGCCCGTCAGCCGCGGCC
GGTGCCAGGATTGCCCCGCAGAGCGCCAGGAGCAGCCCCCACGCCAT
>*Myge-UB*
CATCTGCAGCCCAGGCGCCTGCACTGGGAAGTGGAAGCTGCAGTTGCGGTCTCAGAGCCGGCGTTTCGCTGG
GTACAATCACGGCAAAGGGCGTACCGGAGCTGGCAGAGCCGTCGTGAGCTGAAGCCGCTTTGTAGCCGCCTT
TCTTCTTCCTGAGGCAGAGAACAGCTCCCGCAATGACAGCCACCAGCACGAGAACCCCGAGAACGATCCCCAC
GATCAGCGTCACGCTGGACTTGGGCGCCCAGGGCACTCTCAGATCCTCCGTCAGGCTGGGGTGCTCCACGCA
GCAGGTATAATCCGTCTCTTTGCTGGGGTCGATCTCTATGGTCGCCCGGGTCTGGTAGGTCCCGTCCCCGCTG
GGAACCACTCCCAATTGCATCGTCTCCTGGGGCACGGCCACCCCCTTCTTCAGCCACACGACAGCGACGCTGC
GCGGGTAGAAGCCGTGCACCCGGCAGGAGAGGGTGGTGAGCCCGTCCCCGCTCGGCCGGTCGCTCACCTGC
ACCCGCGGTCGCTTTCCTCTGCAGAGCGTCCTTCCCATACTCCAGGTATTTTTTCAGCCAGTCGATACAGATCTC
CTCCAGGTAATTCTTTCGCCTTTGAAGATGGTCCTTATCAGCCTCCCATTTCCTCTTGGTGACTTGCGCACCAGC
ATCTGCTGCAGTGTACGTCATGGTGTCCTTGTCGAAGCTAACGAAGTCTCTCCCGTCATAGGCATACTGGTGA
AAAGCCCCAGTGGTGCCGTCATCCCGGAGATCACAGCCATACATATTCTGCCAGGTGTGAAAGCCCGCGCTCT
GGTTGTAGCGCCCGCGCAGCGTGTTCAGGTCCACGCGGAAGATGGCCTGCCAGCCCTGCAAGTTCTGCGTCT
CCCGCTCCCAGTACTGCGCGTCCTCGATCCGCGCCGTCCAGTCCGCGCGCGGCTCCGCGCTGCCCCGCGCGCT
GTCGTAGTCCATGAAGCGCTGCCCGTCCACGGACCCCACCGTGATGAAGTCGGGCAGCCCCGGGCCGGGCTC
CGACACGCCCGTGTAGAAATAGCGCAGAGAGTGTGTGCCGGGGGCGCAAAGGGCCCCCGGCAGGGCCACG
GCCCCCAGCAGCAGGAGACGCAGGGCCAGCGCCAT
> *Myge-UC*
TAGCTCCAGTGTCCCAGGCTCCGGGTGTCTGATCCGGATGAAGAGCTGGAGGCAGAATCTTCTCCGATCTTTG
GGCTGCTGGAGCGTAGATGGGTTTGGAGGGGCTGCTGATTTTCTTCTCCACAGGATGACGCTGGCCCCTACG
GCTCCAGCCAGAACCAGCAAGGCCAGGACGATGGTGGCCAGGACCCCAGGGGACAGGGGCCCCTTCTTCCC
AGGGGCCAGGCGAGCGTGGGCTCTGACAGGCTGCTGTGCTCCACCCGGCAGGCGTAGCGGTGCCTGTCCTCC
TGCTGCGGGGAGATCTCCAGGGACGACTGCGTGTAGTAGGTGCCATCGGCGTTGGGCAGGATCCCGCTGGA
GTCTGTCTCCACCAGGATGTCTTCTCCATCCCGCACCCAGGAGACGTGGATGGGACGTGGGTGAAAGCCACT
GACACGGCAGGAGAGGGTGACAGAGCCGCTGGGGGTGTCTCTGCGGGAAACCGAGACCACGGGGGGCACT
GCTGCTCCAGCACCGCCCTCCCCTGCTGCACCAGGCTCTGCAGAGTCCCCAGGCACTCGTGCTGCAGATACTG
CTGGACAAACTTGGTCCAAGTCTTGCCCGTCTCCCAGCGCTGCTTAGGGGCGAAGGCCAGCTGCACGGCCGC
GACCCACGTCCCCGTCTGGTTGTCAAAGCTGATGAAGTCCCTCCCGTTGTAGGCGAACTGAAACCTCGGGTCC
ACGGGGGCCTGGCCGCTCAGTGCACAGCTGACGTGAACCTGCTCGGTGTGAAACCCGCCCGTCTGGTTGTGC
AGCTGCATCCAGCGCCTGGTCTCGACTTTAGAGCCCTCCTCATGCTTCCAGAACTGCTGGGTCTTTTCCTGGAG
ATACTCAACACCCACGGCCTGCGCCACCCACTGCTGGGTGGGTCTGACCTCCCGCATCTCGCTGCTGTAGTAC
GCGATCTTCACGTCATCCAGCTGGGTGATCATGATGAAGTGGTGGGTCCCCTCCTCGTTGATGACTGCTGTGA

CTAGCGAGGCCAGGCTGTGGTGCCTGTCAGCCGCAGCTGCTGCCAGGATTCCCCAGCAGAGCCCCAGGAGAA
GACCCCAGGCCAT
> *Myge-UD*
GATGAAGAGCTGGAGGCAGAATCTTCTCCGGTCTTTGGGCTGCTGGAGCGTAGCCGGGTTTTGGGGGGCTGC
TGATTTCCTTCTCCACAGGATGACGCTGGCCCCTATGGCTCCAGCCAGAACCAGCACGGCCAGGACGATGGCA
GCCAGGACCCCAGGGGACAGGGGCCCCTTCTTCCCAGGGGGCAGGCGAGCGTGGGCTCTGATAGGCTGCTG
TGCTCCACCCGGCAGGCGTAGCGGTGCCTGTCCTCCTGCTGCGGGGAGATCTCCAGGGACGACTGCGTGTAG
TAGGTGCCATCAGCGTTGGGCAGGATCCCGCTGGAGTCTGTCTCCACCAGGATGTTTTCTCCGTCCCGCACCC
AGGAGACGTGGATGGGACGTGGGTGAAAGCCACTGGCGCGGCAGGAGAGTGTGACAGAGCCGCTGGGGGT
GTCTCTGCGGGAAACTGAGACCACAGGAGGTACTGCTGCTCCAGCACCGCCTTCCCCTGCTGCATCAGGCTCC
GCAGGGTCCAGAGGCACTCATGCTGCAGGTACTGCTGGACAAACTGAGTCCAGGTCTTGCCCGTCTCCCAGC
GCTGCTTCTGAAGGAAGGCCGTCTGCACGGCCGCGACCCACGTCCCCGTCTGGTTGTCAAAGCTGATGAAGTC
TCTCCCATCGTAGGCGTACTGGTACCTTGCATCCAGGGGGTCCTGGCCACTCAGGGCACAGCCCACGTGAATC
TGCGAGCTGTGAATCCCACCCGTATGGTTGTGCAGCTGCATCCTCCACCTGGTCCCGCCTTTAAAGCCCTCCTC
GTGCCCCCAGAACTGCTGGGTCTTTTCCTTGATATACTGAGCGCCCACGGCCTGTGCTGCCCACTGCTGGGTG
GGTCTGACCTCTCGCGTGTCGCTGCTGTAGTACGCGATCTTAACATCATCCAGTTCGGCGATCATGATGAAGT
GATAGGTCGCATCTTCGTTGATGATTCCTGTGACTAGCACGGCCAGGCTGTGACGCTCAGCAGCCGCTGCCGC
CCCCAGCAGCGCCCAGCAGAGCCCCAGGGCGACACCCAGGGCCAT
> *Myge-UE*
CACTGCACATCATCTACTGATGCTGGCTGAGCTGTGAGGGGAGAAGGAAGCAGGGTCAGTGTCTCGCAGAAC
GCAGGGTCCCCGTCCTCCCCTGCCCCACACTCGTGACACACGGAGACGCCCCAGTAACGGAGCCGGATGTCG
GTGTTTTGCCGTTGTGCCGGGTACCCAGCGATGGGCGACCTATACGGAGCAGCTGCAGCCGCTTTGTAGCCG
CCTTGAGGCAGAGAACAGCTCCCGCGATGACAGCCGCCAGCACGAGAACCCCGAGAACGATCCCCACGATCA
GCGTCACGCTGGACTTGGGCGCCCAGGGCACTCTCAGATCCTCCATCAGGCTGGGGTGCTCCACGCAGCAGG
TATAATCCGTCTCTTTGCTGGGGTCGATCTCTATGGTCGCCCGGGTCTGATAGGTCCCGTCCCCGCTGGGAAG
CACGTCCCACTGCATTGTCTCCTGGGGCACGGCCACCCCCTTCTTCAGCCACACAACGGCGACGCTGCGCGGG
TAGAAGCCGTGCACCCGGCAGGAGAGGGTGGTGAGCCCGTCCCGGCTTGGCCGGTCGCTCACCTGCACCCGC
GGTTGCTCTCCTCTGCAGCGTCTCCTGCCCATACGCCAGGTACTTCTCCAGCCACTCGATGCAGGTCCCCTCCA
GGTAGGCTCTCCACCCCCGAGTGAAGCTCCTGTCAGCATCCCAGTTCCGTTTGCTGATCTGAGCCGCGTCGTCT
GCCGCTACCCAGGTCTCTCGCTCCTTGTCCAGGCTGAGGAAGTCTCGCCCGTCATACGCCTCCTGGTAAAACCC
CCCGATGGAGCCGTCACCGTGTAGCTCACAGCCGTACATCCTTTGCAGAGTGTGAAATCCCGCGCTGTGGTTG
TAGCGCCCGCGCAGGGTGTTCAGGTTGGCTCGGTACAAGGCCTGCACGCCCTGGGAGACCTGCGTCTCCCCG
GCCCAGTACTGCGCGTCCAGGCTCCGCACCATCCACTCTGCGCGCGGCTCCGCTCTGCGTGTCTCGCTGTCGTA
ATGCAGGATGCGCTGCCCGTCCACGGAGCCGACGATGCTGAACTCGGGCAGCCTCGGGCCGGGCTCCGACAC
CCCCGTGTAGAAATTGCGCAGCGAGTGCAGGCCGGGCCCGGGGGCGCTACGGGCCCCCGGCAGGGCCACGG
CGCCCAGCAGCAGCAGGCGCAGGGCCAGCGCCAT

All exon sequences for MHC class II genes annotated using the reference genome

> *Myge-DAA1*
TCACAAGGGGCCCCGCGGGTTGCGGGCAGCGTTCATCTTCATCCCCTTGATGATGAGGATGGTGCCGGCGAT
GATGCCGATGAGGCCCACGGCCAGGCCCAGGGCGCACACCAGGGTCTCTGTGGTCTCGGGGACGGGGGTGG
GCACCTGGGGCTCCCAGTGCTTCAGGAAGGGCTGGGCCAGCCCCGCGTGCTGCACCCGGCAGTCGTAGAACT
CGTCCTGCCGGGGGATGAAGGGCAGGTAGGAGAACTTGCGGAAGGCGTTGTCCTGGCGGGGGTAGAAGTC
GGTCTCGAAGACGCCCCCCGTCACCTCCTGCCCGTTCTTCAGCCACGTCACACTGAGCGCAGGCGGCGAGAAC
TTGTCCACGAAGCAGATCAGGACGTTGGGCTCGCCCAGCTCCACGGGGCCTTTGGTGAACACGGTCACCTCG
GGGGGCAGTTCTGGGCCCGCGTGTAGTTGGACATCTGGATCATGGTCTCCAGGTTTGCTTTGTCCACAGCGAT
GTTGCCCAGGGCGCCCTGCGCCTCGAAGCTGGTGAAGGTGCCGAAGTCGGGCAGGCGCCAGACGGTCTCCTT
CTTCTCCAGGTCCACGTGGAAGATCTCATCCTGGTCAAACTCGAACATGAACTCCCCGGACTCCCGCTGGGAC
TGGGCCGTGCGCTGGTAGAACTCCGCCTGGGACAGCAGGTTCTCCGTGTCACTGCCCCAGCGCCCAGCAGCG
CCAGCAGGGTGAGCAGGGCCAGCTGGGCCATGGGGACGCCCCGTCCTGCGCCCAT

> *Myge-DAB1*
ATGGGGCCGGGTCGGAGCCTGGGGGCCGGGAGCCGCTGGGCTGGGGCTCTGCTCGTGACCCTGACGGTGCT
GGGAACCCGCCTGGCTCATTGCACGGAGCCCGAAGGGCGGTTCCTGCACCAGGCGAAGCACGACTGTTACTT
CACCAACGGCTCCGAGCGGGTCCGGTTCGTGGATCGGTACATCTACGACCGGCAGCAGATCGTGCACTTCGA
CAGCGACGTGGGGCGGTACGTGGCGGACACGGCGATGGGCCGGCCCACAGCCGAGTATTGGAACAAGGAC
CCGGCCGTGCTGGCGGAGAGGCGGGCCAGGGTGGACACGTTCTGCCGGTACAACTACGGCGTGGCCCAGAC
GGGGAAGGTGGTCGGCCGCGCAGTTCAGCCTGAGGTGACAGTTTTCCCCACCAAATCGGGGTCCCAGCCCCA
CCTGCTGGTTTGCTCCGTGACGGGGTTTTACCCCGGGGAGATCGAGATCAAGTGGCTGAAGAACGGGCAGGA
GCAGACGGCCGGGGTGGTGTCCACGGAGCTGCTCCAGAACGGAGACTGGACCTTCCAGATCCTGGTGATGCT
GGAGACGAGCCCCCGCCGCGGGGACGTCTACGCCTGCCAGGTGCAGCACGTCAGCCTGCCCGAGCCCCTCAC
CGTGCGCTGGGAGGCGCAGTCTGACACCGCCCGGAGCAAGATGCTGACGGGGGTCGGGGGCTTCGTGCTGG
GCCTGATCTTCCTGGTGCCAGGACTGGCCATCTACCTGAGGAACAAGAAAGGGCGCCCCCTTCCCCAACCTGC
AGGGCTCCTGAGTTAG

> *Myge_DAA2*
TCACAAGGGGCCCCGAGGGTTGCGGGCGGCGTTCATCTTCATCCCCTTGATGATGAGGATGGTGCCGGCGAC
GATGCCGATGAGGCCCACGGCCAGGCCCAGGGCGCACACCAGGGTCTCTGTGGTCTCGGGGACCGGGGTGG
GCACCTGGGGCTCCCAGTGCTTCAGGAAGGGCTGGGCCAGCCCCGCGTGCTGCACCCGGCAGTCGTAGAACT
CGTCCTGCCGCGGGATGAAGGGCAGGTAGGAGAACTTGCGGAAGGCGTTGTCCTGGCGGGGGTAGAAGTC
GGTCTCGGAGACGCCCCCCGTCACCTCCTGCCCGTTCTTCAGCCACGTCACGCTGAGCGCGGGCGGGAAGAA
CTTGTCCATGAAGCAGATCAGGACGTTGGGCTCGCCCAGCTCCACGGGGTCTTCGGGGAACACGGTCACCTC
GGGGGGCACGTTCTGGGCCCGCGTGTGGTTGGACCTCTTGATCAGGATCTCCAGGTTGTTCTTGCCCGTAGCC
GCGTTGCCCAGAGCGAACTGTGCATCGAAGCTGGAGAACTCGCCAAATTTGGGCAGGCGCCAGACCGTCTCC
TTCTTCTCCAGGTCCACGTAGAACATCTCATCCTGGTCGAACCCCTGGATGAACTCCCCGGACTCCTGCTGGGA
CTGGGCCGTGCGCTGGTAGAACTCTGCCTGGAAGAGCACGTGCTCCACTTTCACTGCCCCGGTGCCCGGCAG
GGCCAGCAGGGCGAGCAGGGCCAGCTGGGCCATGGGGACGCCCCGTCCTGCGCTCAT

> *Myge_DAB2*
ATGGGGCCGGGTCGGAGCCTGGGGGCCGGGAGCCGCTGGGCTGGGGCTCTGCTCGTGACCCTGACGGTGCT
GAGAACCCGCCTGGCTCATTGCACGGAGCCCGAAGGGCGGTTCGTGCTCCAGTGGAAGGGCGACTGTTATTT
CACCAACGGCACCGAGCGGGTCCGGCTGCTGGGCCGGTACATCTACAACCAGCAGCAGATCGCGCACTTCGA
CAGCGCCGTGGGGCTGTGGGTGCCCGACACGGAGCTGGGCCGGCCCGACGCCGAGTATTGGAACAAGGACC
CGGCCGAGCTGGCGCGCAATCGGGCCGATGTGGACCGGTTCTGCCGGCACAACTACGAGGTGGACGCGCCT
TTCACCGTGGAGCGCAGAGTTCAGCCCAAGGTGAAAGTTTCCCCCACCAAATCGGGGTCCCAGCCCCACGCCC
ACCTGCTGGTTTGCTCCGTGACGGGGTTTTACCCCGGGGAGATCCAGATCACGTGGCTGAAGAACGGGCAGG
AGCAGACGGCCGGGGTGGTGTCCACGGAGCTGCTCCAGAACGGAGACTGGACCTTCCAGATCCTGGTGATG

CTGGAGATGAGCCCCCGGCGCGGGGACGTCTACGCCTGCCAGGTGCAGCACGTCAGCCTGCCCGAGCCCCTC
ACCGTGCGCTGGGAGGCGCAGTCTGACGCCGCCCGGAGCAAGATGCTGACGGGGGTCGGGGGCTTCGTGCT
GGGCCTGATCTTCCTGGCGCCGGGACTGGCCATCTACCTGCGGAACAAGAAAGGGCATCCCCTTCCCCAACCT
GCAGGGCTCCTGAGTTAG

> *Myge_DAA3*
TCACAAGGGGCCCCGCGGGTTGCGGGCGGCGTTCATCTTCATCCCCTTGATGATGAGGATGGTGCCGGCGAT
GATGCCGATGAGGCCCACGGCCAGGCCCAGGGCGCACACCAGCGTCTCTGTGGTCTCGGGGACGGGGGTGG
GCACCTGGGGCTCCCAGTGCTTCAGGAAGGGCTGGGCCAGCCCCGCGTGCTGCACCCGGCAGTCGTAGAACT
CGTCCTGCCGCGGGATGAAGGGCAGGTAGGAGAACTTGCGGAAGGCGTTGTCCTGGCGGGGGTAGAAGTC
GGTCTCGAAGACGCCCCCCACCACCTCCTGCCCGTTCTTCAGCCACGTCATGCTGAGCACGGGCGGGAAGAAC
TTGTCCACGAAGCAGATCAGGACGTTGGGCTCCCCCAGCTCCACGGGGCCTTTGGTGAACACGGTCACCTCG
GGGGGCACGTTCTGGGCCCGCGTGTAGTTGGATCTCTTGATCAGGATCTCCAGGTTTGATTTGTCTGTGGCAA
TGTTGCCCAGGGCGCCCTGTGCCTCGAAGCTGGTGAACTTGCCGAAGTCGGGCAGACGCCAGATGGTCTCCT
TCTTCTCCAGGTCCACGTGGAAGATCTCGTCCTGGTCCAACTCGAACATGAACTCCCCGGACTCCTGCTGGGAC
TGGTCCGTGCGCTGGTAGAACTCCGCCTGGGACAGCAGGTTCTCCGCTGTCACTGCCCCAGTGCCCGGCAGC
GCCAGCAGGGTGAGCAGGGCCAGCTGGGCCATGGGGACGCCCCGTCCTGCGCCCAT

> *Myge_DAB3*
ATGGGGCCGGGTCGGAGCCTGGGGGCCGGGAGCCGCTGGGCTGGGGCTCTGCTCGTGACCCTGTCGGTGCT
GAGAACGCGCCTGGCTCATTGCACGGAGCCCGAAGGCCGGTTCGTGTATCAGTGGAAGCACGAGTGTCAGTT
CACCAACGGCACCGAGCGGGTCCGGCTCCTGCTGCGGTACATCTACAACCAGCAGCAGCTCGCGCACTTCGA
CAGCGACCTGGGGCTGTGGGTGGCGGACACGGAGCTGGGCCGGCCCGACGCCGAGTTTTGGAACAAGGACC
CGGCCGTCCTGGCGCGCAATCGGGCCGATGTGGACCGGTTCTGCCGGCACAACTATGGGGTGGCCCAGACG
GGGAAGATGGTCGGCCGCGCAGTTCAGCCCCAGGTGACAGTTTTCCCCACCAAGTCGGGGTCCCAGCCCCAC
CTGCTGGTTTGCTCCGTGACGGGGTTTTACCCCGGGGGGATCCAGATCAAGTGGCTGAAGAATGGGCAGGAG
CAGACGGCCGGAGTGGTGTCCACGGAGCTGCTCCAGAACGGAGACTGGACCTTCCAGATCCTGGTGATGCTG
GAGATGAGCCCCCGGCGCGGGGACATCTACGCCTGCCAGGTGCAGCACGTCAGCCTGCCCGAGCCCCTCACC
GTGCGCTGGGAGGCGCAGTCTGACGCCGCCCGGAGCAAGATGCTGACGGGGGTCGGGGGCTTCGTGCTGG
GCCTGATCTTCCTGGCGCCGGGACTGGCCATCTACCTGCGGAACAAGAAAGGGCGCCCCGTTCCCCAACCTGC
AGGGCTCCTGCGTTAG

> *Myge_DAA4*
TCACAAGGGGCCCCGCGGGTTGCGGGCGGTGTTCATCTTCATCCCCTTGATGATGAGGATGGTGCCGGCGAT
GATGCCGATGAGGCCCATGGCCAGGCCCAGGGCGCACACCAGGGTCTCTGTGGTCTCGGGGACGGGGGTGG
GCACCTGGGGCTCCCAGTGCTTCAGGAAGGGCTGGGCCAGCCCCGCGTGCTGCACCCGGCAGTCGTAGAACT
CGTCCTGCCGGGGGATGAAGGGCAGGTAGGAGAACTTGCGGAAGGCGTTGTCCTGGCGGGGGTAGAAGTC
GGTCTCGGAGATACCCCCCATCACCTCCTGCCCGTTCTTCAGCCACGTCACGCTGAGCACGGGTGGGAAGAAC
TTGTCCACGAAGCAGATCAGGACGTTGGGCTCGCCCAGCTCCACGGGGCCTTTGGGGAACACGGTCACCTCG
GGGGGATCGTTCGGGGCCCGCGTGCGGTTCAATCTTTTGATCATGATCTCCAGGTTGTTCTTGCCCACGGCCA
TGTTGCTCAGGGCGCCCTGTGCCTCAAAGCTGGCAAACTTGCCAAAGTAGGGCAGGCGCCAGACAGTCTCCTT
CTTCTCCAGGTCCACATGCAAGATCTCGTCCTGGTCGAACTCGAACATGAACTCCTCGGACTCCTGCTGGGATG
GTTCCGTGCGCTGGTAGAACTCCACCTGGGCCGACATGTGTTCCACTGTCACTGCCCCGGTGCCCGGCAGCGC
CAGCAGGGCGAGCAGGGCCAGCTGGGCCATGGGGACGCCCCGTCCTGCGCCCAT

> *Myge_DAB4*
ATGGGGCCGGGTCGGAGCCTGGGGGCCGGGAGCCACTGGGCTGGGGCTCTGCTCGTGACCCTGACGGTGCT
GAGAACGCGCCTGGCTCATTGCACGGAGCCCCCCGAGCATTTCGTGCACCAGGCGAAGTTCGAGTGTCACTTC
ACCAACGGCACCGAGGGGTTCCGGTTCGTGCACCGGCACATCTACAACCGGCAGCAGTTCGCGCACTTCGAC
AGCGACCTGGGGCTGTACGTGGCGGACTCGGAGCTGGGCCGAATCGATGTGGAGACCTGGAACAAGGACCC
GGCCGAGCTGGCGTACCGACGGGCCGGGGCGGAACGGTTCTGCCGGACCAACTACGAGGCGGATGAGCCCT

ACACCATCGACCGCAGAGATCAGCCCGAGGTGACAGTTTCCCCCACCAAATCGGGGTCCCAGCCCCACCCCCA
CCTGCTGGTTTGCTCCGTGATGGGGTTTTACCCCGGGGGGATCCAGATCAAGTGGCTGAAGAACGGGCAGGA
GCAGACGGCCGGGGTGGTGTCCACGGAGCTGCTCCAGAACGGAGACTGGACCTTCCAGATCCTGGTGATGCT
GGAGATGAGCCCCCGGCGCGGGGACGTCTACGCCTGCCAGGTGCAGCACGTCAGCCTGCCCGAGCCCCTCAC
TGTGCACTGGGAGGCGCAGTCTGACGCCGCCCGGAGCAAGATGCTGACAGGTGTTGGGGGCTTCGTGCTGG
GGCTGATCTTCCTGGCGCCGGGGCTGGCCATCTACCTGCGGAACAAGAAAGGGCGCCCCCTTCCCCAACCTGC
AGGGCTCCTGAGTTAG

> *Myge_DAA5*
TCACAAGGGGCCGCGCGGGTTGCGGGCAGCGTTCATCTTCATCCCCTTGATGATGAGGATGGTGCCGGCGAC
GATGCCGATGAGGCCCACGGCCAGGCCCAGGGCGCACACCAGGGTCTCTGTGGTCTCGGGGACGGGGGTGG
GCACCTGGGGCTCCCAGTGCTTCAGGAAGGGCTGGGCCAGCCCCGCGTGCTGCACCCGGCAGTCGTAGAACT
CGTCCTGCCGGGGGATGAAGGGCAGGTAGGAGAACTTGCGGAAGGCGTTGTCGTGGCGGGGATAGAAGTC
GGTCTCGAAGACGCCCCCCGTCACCTCCTGCCCGTTCTTCAGCCACGTCACGCTGAGCGCGGGCGGCGAGAAC
TTGTCCACGAAGCAGATCAGGACGTTGGGCTCGCCCAGCTCCACGGGGCCTTTGGTGAACACGGTCACCTCG
GGGGGCACATTCTGGGCCCACGTGCGGTTGGATCTCTTGATCAGGATCTCCAGGTTTGATTTGTCCGTGGCGA
TGTTGCCCAGGGCGCCCTGCGCCTCAAAGCTGGTGAAGGTGCCGAAGTCGGGCAGGCGCCAGACGGTCTCCT
TCTTCTCCAGGTCCACGTGGAAGGTCTCGTCCTGGTCGAACTCCTGCATGAACTGCCCGGACTCCTGCTGGGA
CTGGGCCGTGCGCTGGTAGAACTCCGCATGGATGTGCACGTGCTCCACTGTGACTGCCCCGGTGCCTGGCAG
GGCCAGCAGGGCGAGCAGGGCCAGCTGGGCCAT

> *Myge_DAB5*
ATGGGGCCGGGTCGGAGCCTGGGGGCCGGGAGCCGCTGGGCTGGGGCTCTGCTCGTGACCCTGACGGTGCT
GAGAACCCGCCTGGCTCATTGCACGGAGCCCCCACGGCGGTTCCTGCACCAGTTCAAGGGCGAGTGTCAGTA
CATCAACGGCACGGAGCGGGTCCGGTTCGTGTACCGGCACATCTACGACCGGCAGCAGATCGCGCACTTCGA
CAGCGACGTGGGCCGGTTCGTGGCGGACACGGAGCTGGGCCGGCCCGACGTGGAGTACTGGAACAACGACC
CGGCCTTACTGGCGGAGAGGCGGGCCGAGGTGGACCGGTTCTGCCGGCACAACTACGGGGTGTACGAGCCT
TTCACCATCGACCGCAGAGTTGAGCCCGAGGTGACAGTTTCCCCCACCAAATCGGCGTCCCAGACCCACCTGC
TGGTTTGCTCCGTGACGGGGTTTTACCCCGGGGGGATCCAGATCAAGTGGCTGAAGAACGGGCAGGAGCAG
ACGGCCGGGGTGGTGTCCACGGAGCTGCTCCAGAACGGAGACTGGACCTTCCAGATCCTGGTGATGCTGGA
GATGAGCCCCCGGCGCGGGGACGTCTACGCCTGCCAGGTGCAGCACGTCAGCCTGCCCGAGCCCCTCACCGT
GCGCTGGGAGGCGCAGTCTGACGCCGCCCGGAGCAAGATGCTGACGGGGGTCGGGGGCTTCGTGCTGGGG
CTGAACTTCCTGGCGCCGGGACTGGCCATCTACCTGCGGAACAAGAAAGGGCTCCTGAGTTAG
